# Nitrate Versus Ammonium Assimilation in *Neochlorella semenenkoi*: ^15^N-Tracking Study of Metabolomic Dynamics

**DOI:** 10.3390/ijms27219497

**Published:** 2026-10-24

**Authors:** Anna Vishnevskaya, Maria Sinetova, Nikita Malyshev, Anastasya Malyukova, Eugene Nikolaev, Yury Kostyukevich

**Affiliations:** 1 Center for Molecular and Cellular Biology, Bolshoy Boulevard 30, bld. 1, Moscow 121205, Russia; ai.vish@yandex.ru (A.V.); nv.malyshev@yandex.ru (N.M.); anastasija.maluckowa@yandex.ru (A.M.); 2 K. A. Timiryazev Institute of Plant Physiology, Russian Academy of Sciences, Moscow 121205, Russia; maria.sinetova@mail.ru; 3 Project Center of Omics Technologies and Advanced Mass Spectrometry, Bolshoy Boulevard 30, bld. 1, Moscow 121205, Russia; ennikolaev@gmail.com

**Keywords:** microalgae, metabolomics, nitrogen starvation, stable isotope labeling, *Neochlorella semenenkoi*, ammonium assimilation, nitrate assimilation, HPLC-MS

## Abstract

Microalgae of the *Chlorellaceae* family are promising producers of valuable metabolites, yet the choice of nitrogen source significantly modulates their physiology and biochemistry. In this study, we investigated the effect of two inorganic nitrogen sources—ammonium acetate (AA) and sodium nitrate—on the metabolic recovery of *Neochlorella semenenkoi* IPPAS C-1210 after nitrogen starvation. Using a combination of targeted amino acid quantification (HPLC-MS/MS) and untargeted high-resolution metabolomics, we tracked both concentration changes and de novo biosynthesis of nitrogen-containing metabolites via ^15^N isotopic labeling. Our results show that ammonium acetate and sodium nitrate enable equally rapid growth resumption and comparable high final biomass yields. Targeted analysis revealed that ammonium-fed cells restored their amino acid pools substantially within 1 day, with a pronounced accumulation of glutamate and glutamine, whereas nitrate-fed cells exhibited a slower and only partial recovery of their free amino acid pool, with several amino acids remaining undetectable even after 3 days. Untargeted metabolomics identified 308 unique metabolites, and the heatmaps of the most variable compounds highlighted a marked early upregulation of TCA cycle intermediates, stress-signaling molecules, and nucleotide precursors in AA cultures, in contrast to a delayed, lipid-centric response in nitrate cultures. The ^15^N tracing confirmed near-complete (>96%) enrichment of amino acids in both sources, demonstrating that recovery is driven by de novo synthesis rather than recycling, and that isotopic enrichment extends to nucleotides and the NAD^+^ precursor nicotinamide. These findings reveal the metabolic advantages of ammonium over nitrate for rapid metabolic recovery and restoration of nitrogen-rich compound pools, and provide a foundation for optimizing cultivation strategies for this biotechnologically relevant alga.

## 1. Introduction

Microalgae, especially representatives of the *Chlorellaceae* family, are promising producers of biologically active compounds, including lipids, proteins, and various metabolites of secondary origin [1,2]. The ability of these microorganisms to synthesize valuable substances largely depends on the cultivation conditions, among which the nature of nitrogen nutrition plays a key role. Nitrogen is a critical element that is part of amino acids, nucleic acids, chlorophylls, ATP/ADP, and many other important compounds, and its availability and chemical form directly affect the physiological and biochemical state of cells [3,4].

Changes in growth conditions, in particular the introduction of stress factors such as nitrogen starvation, can radically restructure the metabolism of microalgae, stimulating the accumulation of reserve lipids and carbohydrates. This process is an adaptive mechanism that allows cells to survive in conditions of resource scarcity. In *Chlorella vulgaris*, nitrogen stress has been shown to induce a two-stage response: at the first stage, carbohydrates accumulate and proteins degrade, and at the second stage, lipids, in particular triacylglycerols (TAGs), actively accumulate [5,6]. However, the details of this metabolic switch vary significantly between different strains and depend on their genetic characteristics, as well as on the form of available carbon and the chemical form of nitrogen supplied [7,8].

Among the inorganic nitrogen sources used for microalgae cultivation, nitrates (NO_3_^−^) and ammonium (NH_4_^+^) are the most common. However, the metabolic pathways of their assimilation are fundamentally different, which makes the choice of a nitrogen source critically important for cell physiology. Classical studies have shown that *Chlorella* is able to assimilate nitrogen from both nitrate and ammonium; however, in the simultaneous presence of both sources, cells preferentially use ammonium, and nitrate assimilation is suppressed [9,10]. Morris and Syrett demonstrated that *Chlorella vulgaris* grown on ammonium does not assimilate nitrate, but acquires this ability after nitrogen starvation, and the activity of nitrate reductase in such cells is initially very low, but increases during starvation [11]. It was also found that the addition of ammonium causes the complete cessation of nitrate assimilation, which resumes only after the depletion of ammonium from the medium [12]. In this case, the inhibition is due not to ammonium itself, but to the products of its assimilation [12].

Assimilation of nitrate is an energy-consuming process requiring its sequential reduction to nitrite and then to ammonium. This process involves the work of the nitrate reductase enzyme, which catalyzes the limiting step, the reduction of nitrate to nitrite [13], and consumes a significant amount of reducing equivalents. It has been shown that up to 20% of the energy stored during photosynthesis in *Chlorella* sp. can be spent on nitrate reduction [14]. Unlike nitrate reduction, ammonium assimilation is less dependent on light and can use both replacement endogenous products and newly synthesized photosynthetic products [14]. Ammonium is a more reduced form of nitrogen and can be directly incorporated into organic compounds through the key enzymes glutamine synthetase (GS) and glutamate synthase (GOGAT), which requires lower energy costs [15]. For *Chlorella protothecoides*, it has been shown that under photoautotrophic conditions, ammonium is assimilated primarily through the glutamine synthetase pathway, whereas under mixotrophic and heterotrophic conditions, both NADH- and NADPH-dependent glutamate dehydrogenase pathways are induced [16].

These metabolic differences lead to the fact that the choice of a nitrogen source can have different effects on the growth, biochemical composition of biomass, and ultimately on the productivity of microalgae by target compounds. For example, for the microalgae *Mychonastes afer*, it has been shown that different forms of nitrogen (ammonium, nitrate, urea) have different effects on the accumulation of triacylglycerols and the composition of fatty acids [17]. In the work of Lachmann et al. (2019), it was demonstrated that green algae *Chlamydomonas acidophila* grown on ammonium had higher carbon fixation rates compared to cells on nitrate; however, the accumulation of more amino acids on nitrate was observed [3]. Studies on *Chlorella sorokiniana* have shown that nitrogen concentration and its source significantly affect the lipid content, which is important for biofuel production [18]. For *Nannochloropsis oceanica*, it was found that cells grown on ammonium sulfate and ammonium nitrate produced the highest content of total lipids (65% and 62% of dry weight, respectively), with the fatty acid profile significantly varying depending on the nitrogen source [19]. At the same time, it has been shown for some *Chlorella* strains that ammonium nitrogen and nitrogen starvation can have an inhibitory effect on the growth and accumulation of lipids [20].

In recent years, new, previously undescribed strains of *Chlorella*-like algae with unique biotechnological properties have been actively studied. One of such promising objects is *Neochlorella semenenkoi* IPPAS C-1210 [21]. *Neochlorella semenenkoi* IPPAS C-1210 is a freshwater green alga of the family Chlorellaceae. In our previous work, it was shown that this strain is able to grow efficiently on a modified BG-11 medium with ammonium acetate as the only nitrogen source, successfully utilizing the ammonium form [22]. Changes in metabolism and lipid composition of cells during nitrogen starvation and subsequent recovery were characterized in detail using isotope labeling of ^15^N and high-performance liquid chromatography-mass spectrometry (HPLC-MS). It has been demonstrated that the percentage of isotopic label inclusion in compounds often differs significantly from their quantitative changes, which highlights the advantage of isotope analysis for studying the dynamics of metabolic fluxes [22]. Isotopic labeling of ^15^N in combination with high-resolution mass spectrometry has been successfully used to study the nitrogen metabolism of microalgae, making it possible to track the pathways of nitrogen incorporation into various classes of compounds and evaluate the rates of metabolic processes in vivo [23,24].

However, the comparative effect of the two main inorganic nitrogen sources, sodium nitrate and ammonium acetate, on the metabolism and lipidomics of *Neochlorella semenenkoi* IPPAS C-1210 remained unexplored. Thus, for the first time for N. semenenkoi IPPAS C-1210, we directly compare sodium nitrate and ammonium acetate as nitrogen sources during recovery from nitrogen starvation using targeted amino acid quantification, untargeted high-resolution metabolomics, and ^15^N isotopic tracing. This integrated approach distinguishes concentration changes from de novo biosynthetic fluxes and reveals source-specific metabolic reprogramming. Given the fundamental differences in energy costs for the assimilation of these two forms of nitrogen [9,14], their known effect on the enzymatic apparatus of the cell [11,12] and the accumulation of lipids in other strains of microalgae [17,18,19], it can be expected that the choice of a nitrogen source will significantly modulate intracellular metabolic fluxes, the direction of biosynthetic processes and, ultimately, the composition of biomass. Understanding these mechanisms is crucial for optimizing cultivation conditions for the targeted synthesis of target products such as lipids for biofuels or other valuable metabolites.

In this paper, we present the results of a comprehensive amino acid and metabolomic analysis of *Neochlorella semenenkoi* IPPAS C-1210 grown in the presence of two different nitrogen sources: sodium nitrate (NaNO_3_) and ammonium acetate (NH_4_COOCH_3_). Using the ^15^N isotope labeling methodology in combination with high-resolution HPLC-MS, we for the first time evaluate and compare the dynamics of nitrogen incorporation into a wide range of nitrogen-containing compounds, including amino acids, nucleotides and other metabolites. Our work is aimed at identifying the metabolic features caused by the nature of the nitrogen source, which will allow us to better understand the physiology of this promising strain and develop strategies for its effective biotechnological application.

## 2. Results

### 2.1. Growth Characteristics Under Different Nitrogen Sources

**Figure 1:**
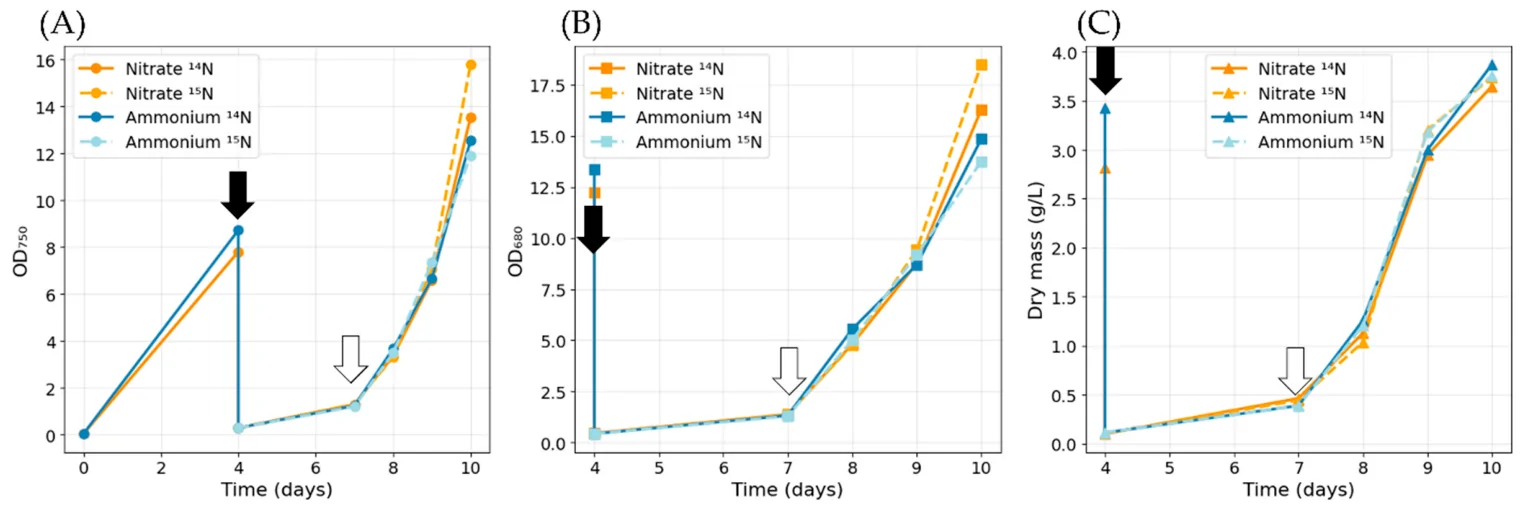
Growth characteristics of *Neochlorella semenenkoi* IPPAS C-1210 with different nitrogen sources and isotopic labels. (**A**) Optical density at 750 nm (OD_750_) over the experimental timeline. (**B**) Optical density at 680 nm (OD_680_) over the period from day 4 to day 10. (**C**) Dry biomass accumulation (g/L) over the same timeline. Days 0–4: growth on full medium with respective nitrogen source; days 4–7: nitrogen starvation; days 7–10: recovery with ^14^N- or ^15^N-labelled ammonium acetate or sodium nitrate. Symbols: circles, OD_750_; squares, dry mass. Solid lines: ^14^N; dashed lines: ^15^N. Colours: blue—ammonium acetate; orange—sodium nitrate. Black arrow, transfer to nitrogen-free medium; white arrow, addition of nitrogen sources.

To assess the physiological state and overall fitness of *Neochlorella semenenkoi* IPPAS C-1210 under different nitrogen regimes, we monitored cell growth by measuring optical density at 680 and 750 nm (OD_680_ and OD_750_, respectively) and dry biomass accumulation over the entire experimental timeline (Figure 1A–C). OD_750_ reflects biomass concentration, whereas OD_680_ depends on both biomass concentration and chlorophyll *a* content.

The cultures were grown for 4 days prior to nitrogen starvation (days 0–4), then subjected to 3 days of nitrogen starvation (days 4–7), and finally replenished with either ^14^N- or ^15^N-labelled ammonium acetate or sodium nitrate (days 7–10).

During the initial growth phase (days 0–4) on full medium, both cultures showed exponential growth with similar OD_750_ and OD_680_ values, regardless of the nitrogen source (Figure 1A,B). Upon transfer to nitrogen-free medium (day 4), growth immediately slowed down in all cultures. Over the 3 days of nitrogen deprivation, OD_750_ increased only moderately, from ~0.28 to ~1.2–1.3 (a ~4.3-fold increase), while OD_680_ rose even less in relative terms, from ~0.43 to ~1.3–1.4 (a ~3-fold increase), reflecting chlorosis and confirming effective nitrogen starvation. Consistent with OD_750_ change, the biomass concentration increased from 0.1 to 0.39–0.46 g/L (Figure 1C).

After nitrogen replenishment at day 7, all cultures immediately resumed growth with kinetics indistinguishable between ammonium acetate and sodium nitrate, and likewise between ^14^N and ^15^N treatments (Figure 1A,B). Similarly, the dry biomass accumulation (Figure 1C) reached final values of ~2.8–3.0 g/L in all cultures by day 10, with no significant differences between the conditions. These results demonstrate that *N. semenenkoi* efficiently utilises both ammonium and nitrate as nitrogen sources, with comparable growth kinetics and final biomass yields. The absence of any isotopic effect on growth confirms that ^15^N labelling does not perturb the overall physiology of the cells, validating its use for metabolic tracing.

### 2.2. General Dynamics of Amino Acid Pools During Starvation and Recovery

To monitor the metabolic response of *Neochlorella semenenkoi* IPPAS C-1210 to nitrogen replenishment after starvation, we performed a semi-quantitative analysis of 17 proteinogenic amino acids using HPLC-MS/MS with external calibration curves (Supplementary Figures S1–S17). The calibration curves showed excellent linearity with R^2^ > 0.95 for all amino acids, allowing reliable conversion of peak areas into concentrations (µg per mg of lyophilized biomass). The extraction procedure and chromatographic conditions are described in the Materials and Methods section. We quantified the total intracellular pools of amino acids under two nitrogen sources—ammonium acetate (AA) and sodium nitrate—at five time points: before starvation (after 4 days of growth on full medium), after 3 days of nitrogen starvation, and at 1, 2, and 3 days after the addition of either an unlabeled (^14^N) or isotopically labeled (^15^N) nitrogen source. The quantitative results of the amino acid analysis are compiled in Table 1.

**Figure 2:**
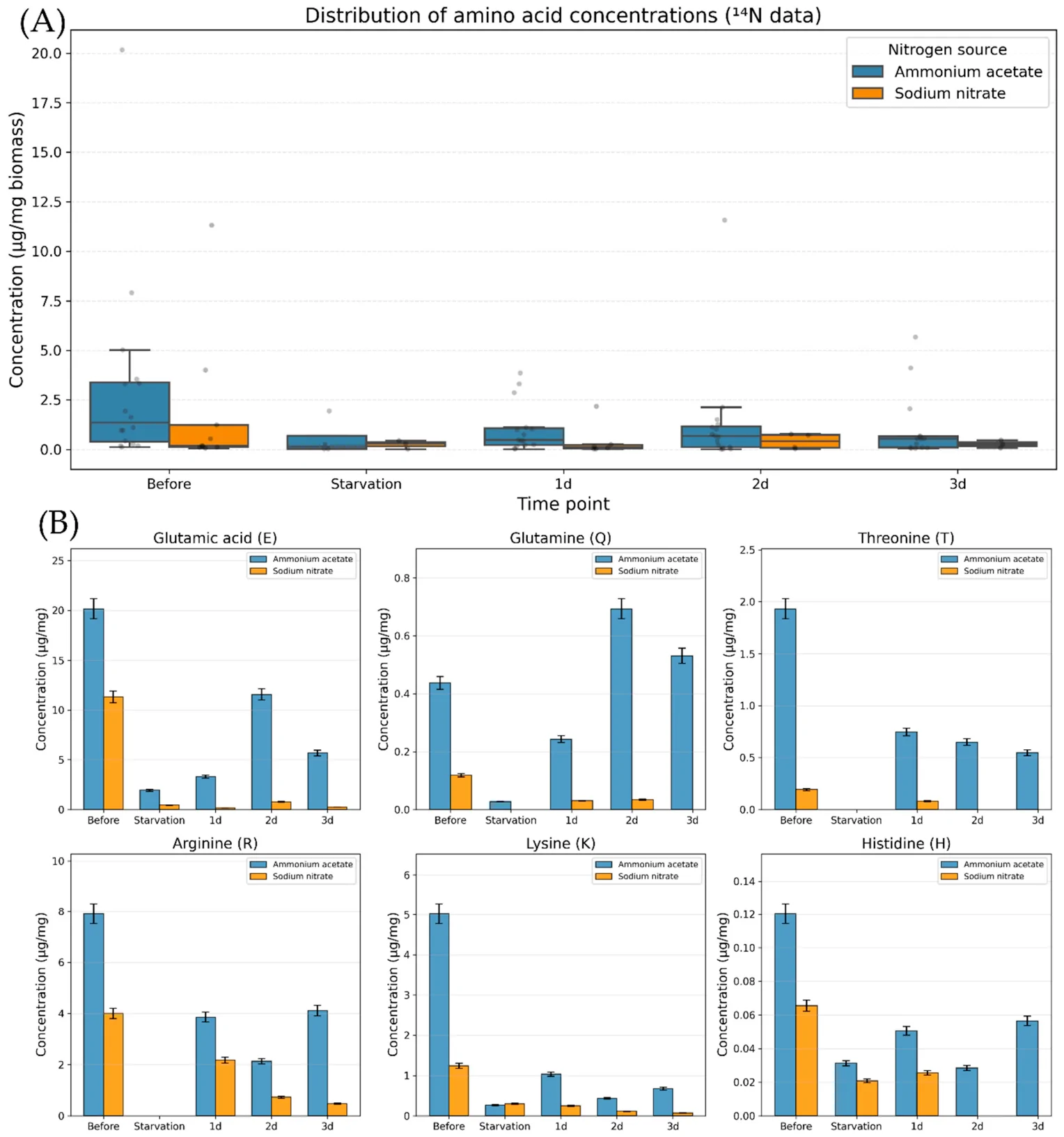
(**A**) Boxplots showing the distribution of mean ± SD (of three biological replicates, *n* = 3) concentrations across all quantified amino acids at each time point (Before, Starvation, 1 d, 2 d, 3 d) for ^14^N data. The boxes represent the interquartile range (IQR), the horizontal line indicates the median, and the whiskers show the range (1.5 × IQR). Individual points represent single amino acids. Ammonium acetate significantly elevated the median amino acid pool on day 1 compared to nitrate (Mann–Whitney U test, U = 168.00, *p* = 0.0047, FDR-adjusted *p* = 0.0141). (**B**) Bar plots (mean ± 5% error) for selected key amino acids: glutamate (E), glutamine (Q), threonine (T), arginine (R), lysine (K), and histidine (H). Complete individual data for all quantified amino acids are provided in Table 1.

Nitrogen starvation caused a pronounced decrease in the concentration of most amino acids (Figure 2A). Notably, even before starvation, the total amino acid pool in nitrate-grown cells was substantially lower than in ammonium-grown cells (approximately 17.8 vs. 51.1 µg/mg biomass, respectively; Table 1). Upon addition of either ammonium acetate or nitrate, the amino acid pools recovered, although the kinetics and extent of recovery differed markedly between the two nitrogen sources. For cultures supplemented with ammonium acetate, the total amino acid content increased within 1 day, reaching approximately 31% of the pre-starvation level (16.0 µg/mg vs. 51.1 µg/mg before starvation), reached a maximum on day 2 (21.7 µg/mg, ~42%), and then declined on day 3 (15.5 µg/mg, ~30%). The decline on day 3 likely reflects the utilization of newly synthesized amino acids for protein synthesis and other biosynthetic processes, rather than continued accumulation in the free pool. In contrast, nitrate-fed cultures exhibited a slower and only partial recovery: by day 1, the total amino acid pool remained significantly lower than in ammonium-fed cells, and even by day 3, several amino acids (e.g., proline, valine, leucine, isoleucine, asparagine, phenylalanine) were still not detected (Table 1, Figure 2A). These observations suggest that ammonium, being a directly assimilable form, enables a faster and more extensive recovery of the free amino acid pool compared to nitrate, which requires prior reduction to ammonium. However, in both cases the free amino acid pool remained below pre-starvation levels, indicating that a substantial fraction of the newly assimilated nitrogen was rapidly channeled into macromolecules rather than accumulating as free amino acids. Heatmaps of individual amino acid concentrations in ammonium acetate and sodium nitrate cultures are provided in Supplementary Figures S18 and S19.

The amino acids shown in Figure 2B showed the most pronounced differences between the two nitrogen sources during early recovery. Note the rapid accumulation of glutamate and glutamine in ammonium acetate cultures on day 1, consistent with primary ammonium assimilation via the GS-GOGAT pathway. In contrast, nitrate cultures exhibited delayed and reduced accumulation, with many amino acids (e.g., proline, valine, leucine) remaining undetectable on day 1 (Table 1).

### 2.3. Differential Recovery Between Ammonium and Nitrate Sources

**Figure 3:**
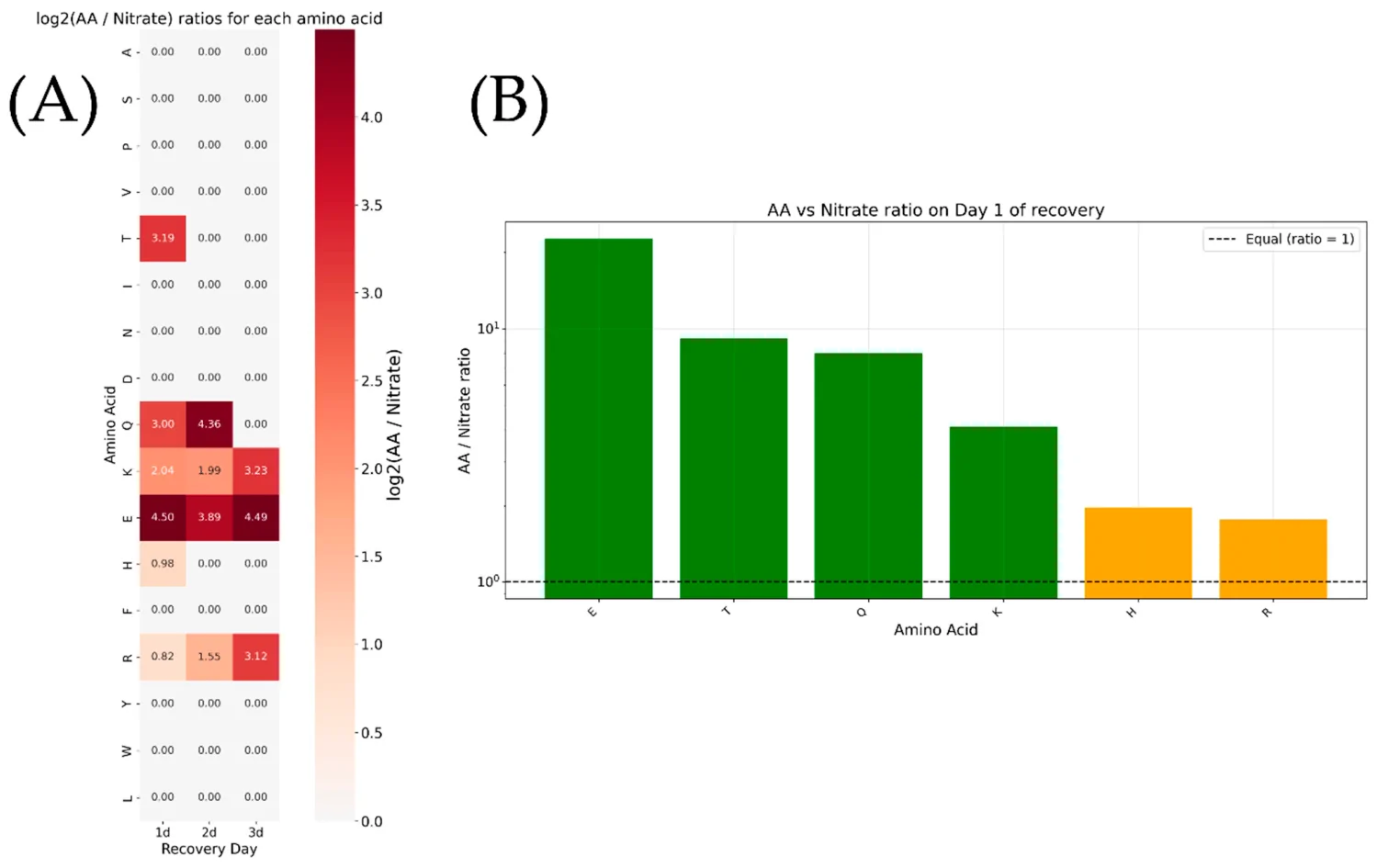
(**A**) Heatmap of log_2_(AA/Nitrate) ratios for each amino acid across the three recovery days (1 d, 2 d, 3 d) based on ^14^N data. Positive values (red) indicate higher concentrations in AA cultures. Values of 0.00 in the heatmap indicate that the corresponding amino acid was not detected (below the limit of quantification) in the nitrate-fed culture, while it was clearly present in the AA culture. (**B**) Bar plot of the day-1 ratios (log scale); the dashed line marks ratio = 1.

To quantitatively compare the efficiency of the two nitrogen sources, we calculated the ratio of concentrations in AA-fed cells to those in nitrate-fed cells for each amino acid and each recovery day using only the ^14^N data (because the ^15^N variant had only one biological replicate (n = 1), only ^14^N data (n = 3) were used for the AA-versus-nitrate comparison). The log_2_-transformed ratios are presented as a heatmap in Figure 3A. On day 1, the majority of amino acids showed higher concentrations in AA cultures, with the most pronounced differences observed for glutamate (E, log_2_ = 4.50), threonine (T, log_2_= 3.19), glutamine (Q, log_2_ = 3.00), lysine (K, log_2_= 2.04), and arginine (R, log_2_= 0.82). Importantly, several amino acids—including proline (P), valine (V), leucine (L), asparagine (N), phenylalanine (F), and isoleucine (I)—were not detectable in nitrate-fed cells on day 1, whereas they were clearly present in AA-fed cells. In the heatmap, these cases appear as 0.00 (see legend), reflecting the complete absence of these amino acids in the nitrate condition rather than equal concentrations.

By day 2, the ratios decreased substantially for most amino acids. By day 3, they approached unity (log_2_ ≈ 0) for the amino acids that were detectable in both conditions (glutamate, glutamine, lysine, arginine, threonine, histidine). However, several amino acids—including proline, valine, leucine, isoleucine, asparagine, and phenylalanine—remained undetectable in nitrate cultures even on day 3 (Table 1, Figure 3A). Thus, while nitrate cultures showed partial recovery of a limited set of amino acids—with increasing concentrations of glutamate, glutamine, lysine, arginine, and threonine—they did not achieve the full complement of amino acids observed in AA cultures within the 3-day recovery window. This temporal pattern indicates that ammonium acetate enables the rapid restoration of a broad spectrum of free amino acids, whereas nitrate leads to a delayed and partial recovery of only a subset of amino acids within the 3-day recovery window.

The day-1 ratios are further illustrated in Figure 3B as a bar plot on a logarithmic scale, where the dashed line marks ratio = 1; amino acids with undetectable levels in nitrate are shown as the highest bars (with values capped at the maximum finite ratio for visualization), clearly demonstrating the immediate advantage of ammonium acetate during the early recovery phase.

**Figure 4:**
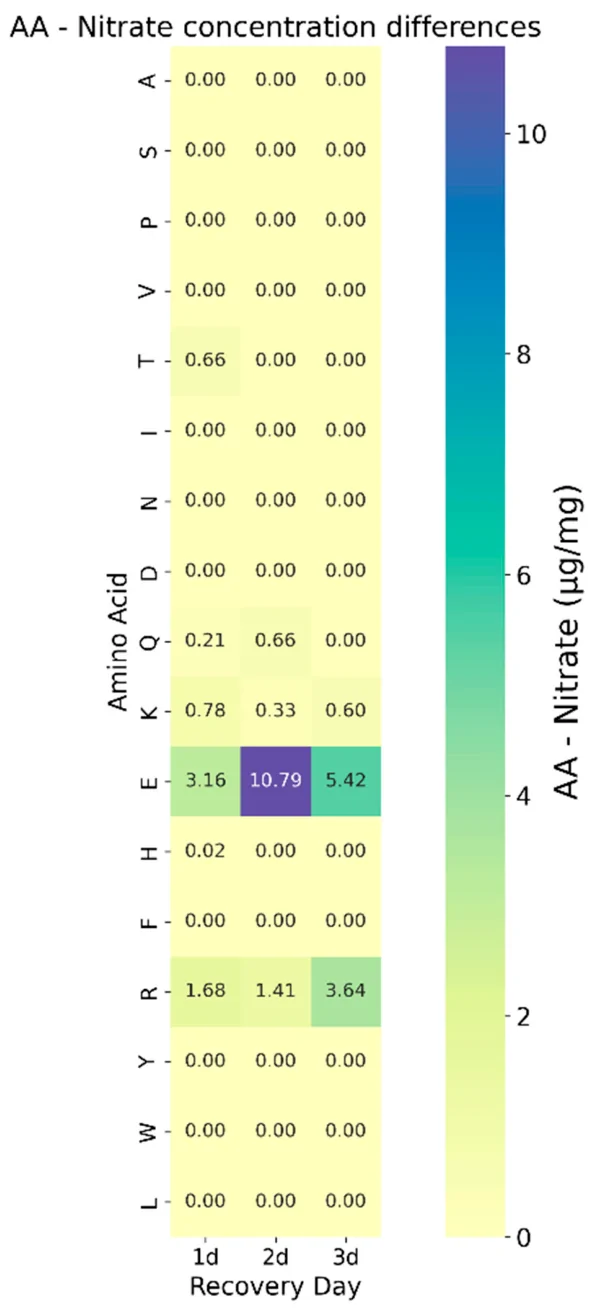
Heatmap of absolute concentration differences (AA—Nitrate, µg/mg biomass) for each amino acid across recovery days. Positive values (yellow to blue) indicate higher concentrations in AA cultures. The data shown are based on ^14^N measurements.

The absolute concentration differences (AA—Nitrate) are shown in Figure 4. Glutamate exhibited the largest difference (3.16 µg/mg on day 1), followed by arginine (1.68), lysine (0.78), threonine (0.66), glutamine (0.21), and histidine (0.02). These data confirm that the most prominent effect of ammonium is on the glutamate/glutamine family, which is consistent with the central role of these amino acids in primary nitrogen assimilation via the GS-GOGAT pathway [4,25].

### 2.4. Statistical Comparison of Ammonium and Nitrate on ^14^N Cultures

To assess whether the observed differences between ammonium acetate and nitrate cultures were statistically significant, we performed Mann–Whitney U tests comparing the concentrations of all amino acids (pooled across the 17 detected amino acids) for each recovery day using only the ^14^N data. The Mann–Whitney U test was chosen because the data were non-normally distributed and contained numerous values below the limit of quantification (particularly in nitrate cultures), which violate the assumptions of parametric tests such as the t-test. Values marked as <LOQ or ND were excluded from the analysis and were not treated as zero. The pooled analysis across amino acids is used here as a summary measure of the total free amino acid pool. It does not replace metabolite-specific analysis, which is presented in Table 1 and Figure 2B, Figure 3 and Figure 4.

The results are summarised in Table 2. On day 1, the difference was statistically significant after correction for multiple comparisons (U = 168.00, *p* = 0.0047, FDR-adjusted *p* = 0.0141). On day 2 (U = 115.50, *p* = 0.0142, adjusted *p* = 0.0159) and day 3 (U = 87.00, *p* = 0.0159, adjusted *p* = 0.0159), the differences also remained significant.

This confirms that ammonium acetate significantly elevates the amino acid pool compared to nitrate throughout the recovery period. The decrease in U-statistic from day 1 to day 3 suggests that the magnitude of the difference gradually diminishes, consistent with a progressive convergence of the amino acid profiles, although the convergence is not yet complete by day 3 (see also Table 1 and Figure 2A).

The pooled analysis across amino acids is used here as a summary measure of the total free amino acid pool. It does not replace metabolite-specific analysis, which is presented in Table 1, Figure 2B, Figure 3 and Figure 4. For amino acids with the strongest differences, individual concentration data and ratios are reported separately.

### 2.5. Absence of Isotopic Effect: Comparison of ^14^N and ^15^N Cultures

To verify that the substitution of ^15^N for ^14^N does not introduce a bias in the overall amino acid pools, we compared the total concentrations of amino acids in ^14^N- and ^15^N-supplemented cultures for each nitrogen source and each recovery day. Because the 15N variant was performed in a single cultivation vessel (n = 1), while the ^14^N variant had three biological replicates (n = 3), formal statistical testing of ^14^N versus ^15^N was not possible. We therefore present a descriptive comparison.

For ammonium acetate cultures, the total amino acid concentrations in the ^15^N samples were close to the ^14^N means and fell within the ^14^N mean ± SD on all recovery days (Table 3). For nitrate cultures, only day 1 could be compared because on days 2 and 3, most amino acids were below the limit of quantification in the ^15^N samples. On day 1, the ^15^N value was also close to the ^14^N mean. No consistent direction of deviation was observed between the two isotopic variants. Absolute concentrations in ^15^N samples differed from 14N means for several amino acids; because ^15^N had *n* = 1, these differences cannot be formally attributed to either an isotopic effect or biological variability. The conclusion that recovery is driven by de novo synthesis rests on isotopic enrichment (Table 4), not on concentration equivalence.

These data indicate that ^15^N labelling did not cause a pronounced perturbation of amino acid pools. However, because of the single biological replicate for ^15^N, we cannot formally establish statistical equivalence. The comparison should therefore be considered descriptive and indicative rather than definitive.

These results indicate that isotopic labeling does not affect the metabolic turnover or the steady-state concentrations of amino acids. This finding validates the use of ^15^N labeling for future flux studies, as the label faithfully reports de novo synthesis without perturbing the physiology of the cells.

### 2.6. Isotopic Labeling Patterns and Dynamics of De Novo Amino Acid Synthesis

To directly assess the contribution of newly assimilated nitrogen to the amino acid pools during recovery, we analyzed the incorporation of the ^15^N isotope into amino acids. For each amino acid detected in the ^15^N-supplemented cultures, we calculated the percentage of labeled molecules (^15^N enrichment) as the ratio of the ^15^N-containing isotopologue peak area to the total (^14^N + ^15^N) peak area. The full dataset is summarised in Table 4, which presents the enrichment percentages for all detectable amino acids across the three recovery days, separately for each nitrogen source. It should be noted that the values in Table 4 are reported only for amino acids that were detectable in the corresponding samples (as indicated in Table 1); for samples where an amino acid was not detected, the enrichment is marked as n.d.

As shown in Table 4, ^15^N enrichment exceeded 96% for all detectable amino acids in both nitrogen sources, with the glutamate/glutamine family showing 97–100% enrichment. This near-complete labelling indicates that the free amino acid pools after nitrogen replenishment were dominated by de novo synthesized molecules rather than recycled pre-existing pools. To test whether the ^15^N enrichment changed significantly over time within each source, we calculated the standard deviation across the recovery days for which data were available. The remarkably low standard deviations (typically <2%) confirm that the isotopic labeling reached a steady state rapidly and remained stable throughout the recovery period in both sources.

Importantly, these near-complete labeling levels indicate that the vast majority of free amino acids present in the cells after nitrogen replenishment were synthesized de novo from the newly added ^15^N-labeled nitrogen source (either ammonium or nitrate), rather than being recycled from pre-existing pools. This conclusion is consistent with the rapid depletion of amino acids during starvation (Table 1) and their subsequent rebuilding from fresh nitrogen. The especially high labeling in the glutamate/glutamine family (97–100%) is in full agreement with the central role of these amino acids in primary nitrogen assimilation via the glutamine synthetase/glutamate synthase (GS-GOGAT) pathway [4,25].

Notably, although the 15N enrichment remained high and stable across all three days for both sources, the absolute concentrations of free amino acids were highest before starvation (51.1 μg/mg in ammonium and 17.8 μg/mg in nitrate), partially recovered on day 1 (16.0 and 2.7 μg/mg, respectively), and gradually declined thereafter (15.5 and 0.8 μg/mg on day 3) (Table 1). This suggests that the early recovery phase is characterized by a burst of de novo biosynthesis, followed by a period of utilization of these newly synthesized amino acids for protein synthesis and other metabolic processes, rather than continued accumulation in the free pool.

**Figure 5:**
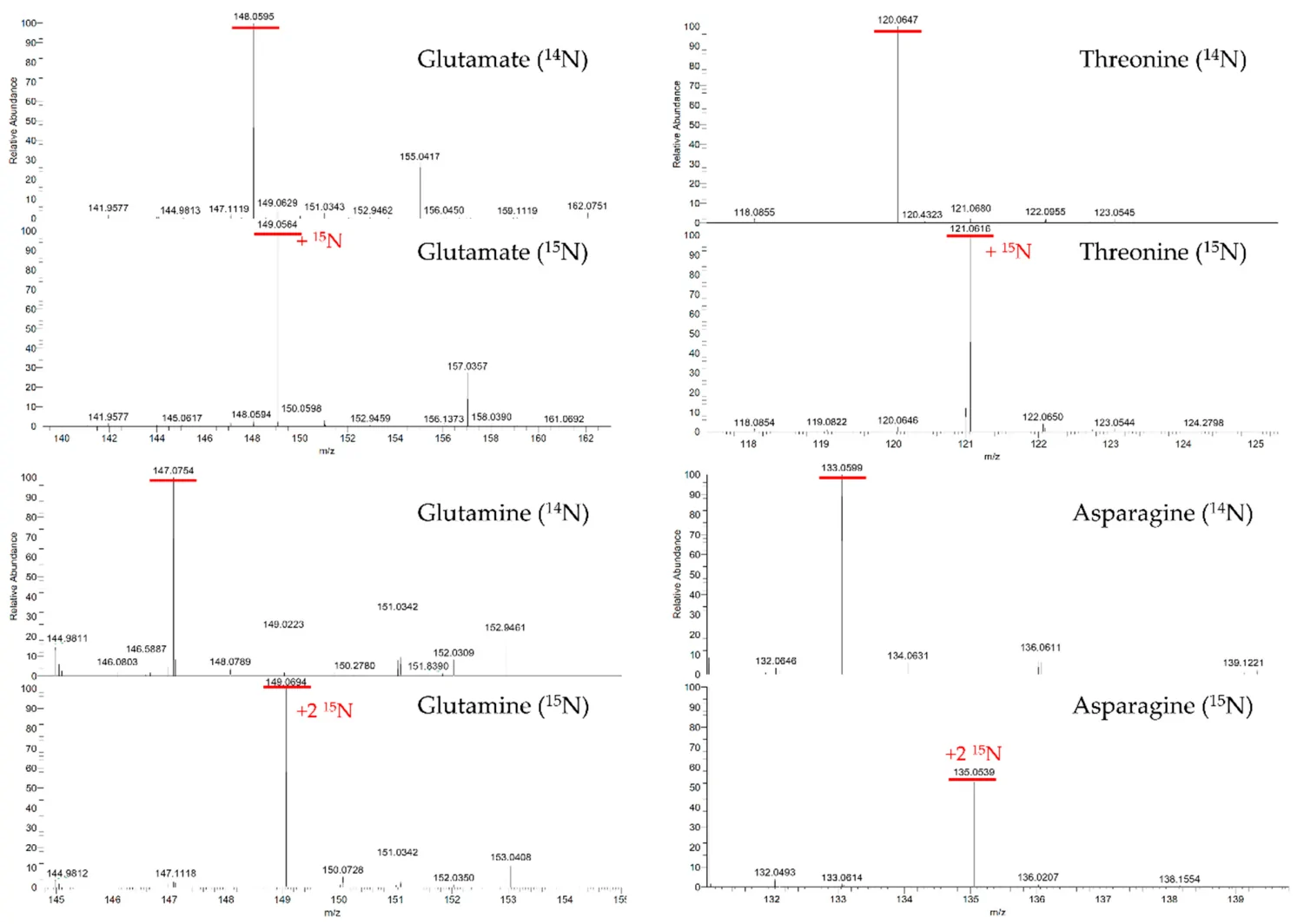
Representative mass spectra of selected amino acids in unlabeled (^14^N) and ^15^N-labeled cultures 24 h (1 day) after nitrogen replenishment.

Representative mass spectra of selected amino acids in ^14^N and ^15^N cultures are shown in Figure 5, demonstrating the almost complete replacement of ^14^N by ^15^N in the newly synthesized pools.

### 2.7. Untargeted Metabolomics Reveals Global Metabolic Reprogramming

To complement the targeted amino acid analysis, we performed untargeted high-resolution metabolomics on the same polar (upper aqueous) extracts used for amino acid quantification. After stringent filtering and removal of synthetic or non-biological compounds, 308 unique metabolites were putatively annotated (annotation score ≥ 70% against mzCloud and MassBank). Because the analysis was performed on the aqueous phase, the annotated metabolites represent polar and semi-polar compounds; intact non-polar lipids were not measured in this study. These metabolites were assigned to 10 major classes (Table 5), with the largest groups being organic acids (57), amino acids and their derivatives (60), lipids and fatty acids (33), nucleotides and bases (26), and carbohydrates (19). The remaining compounds were classified as terpenes (5), vitamins and cofactors (8), steroids (1), and flavonoids (1). A substantial number of metabolites (98) could not be assigned to any of the above classes and were grouped as ‘Other (unclassified)’; these include many low-abundance or poorly annotated features that may represent novel or less-studied compounds. Of the 308 identified metabolites, 296 exceeded the detection threshold (peak area > 1000) in at least one of the two nitrogen sources; the remaining 12 metabolites were below this threshold in both conditions and are listed in Supplementary Table S1.

To investigate the condition-specific metabolic landscapes, we compared the metabolite profiles of ammonium acetate (AA) and sodium nitrate (Nitrate) cultures. Using a detection threshold of 1000 peak area units (to distinguish signal from noise), we found that AA cultures contained 250 detectable metabolites, while Nitrate cultures contained 132 (Table 6). Among these, 86 metabolites were common to both sources, 164 were unique to AA, and 46 were unique to Nitrate. This indicates that ammonium acetate supports a broader metabolic repertoire, likely reflecting its more direct role in nitrogen assimilation and the production of nitrogen-rich secondary metabolites. The complete lists of unique metabolites for each nitrogen source, along with their *m/z* values and metabolic classes, are provided in Supplementary Table S2.

**Figure 6:**
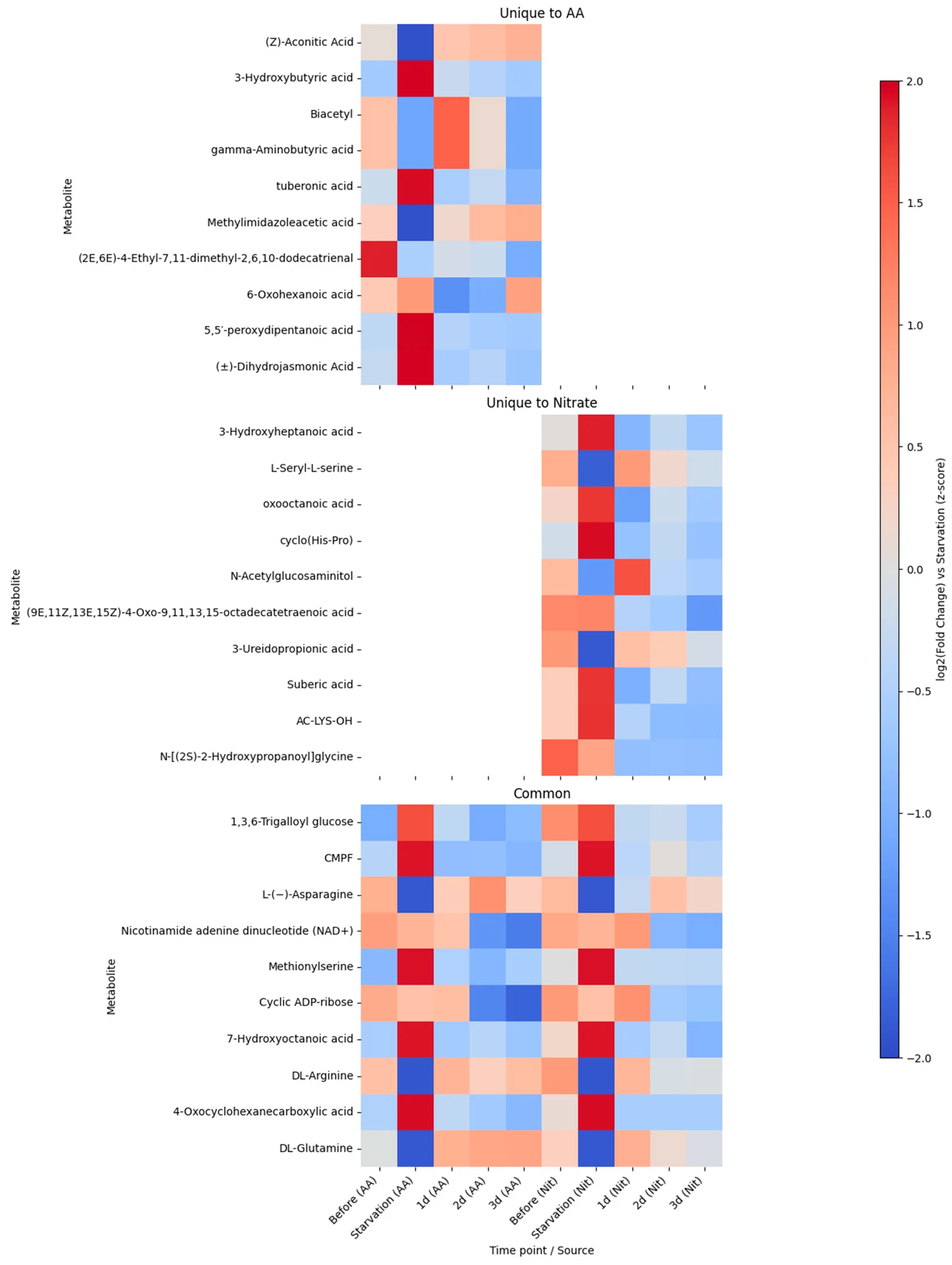
Heatmaps of the top 10 most variable metabolites for each category: unique to ammonium acetate (AA), unique to Nitrate, and common to both sources. For each metabolite, the log_2_ fold change relative to the starvation state is shown for both AA (**left half**) and Nitrate (**right half**) cultures across the five time points (Before, Starvation, 1 d, 2 d, 3 d). Red indicates upregulation, blue indicates downregulation, and white cells indicate that the metabolite was not detected in that particular sample (peak area below 1000). The color bar on the right indicates the z-score-normalised log_2_ fold change.

To visualise the temporal dynamics of metabolite accumulation during recovery, we generated separate heatmaps for the top 10 most variable metabolites in each of three categories: unique to ammonium acetate (AA), unique to sodium nitrate (Nitrate), and common to both sources (Figure 6). For each metabolite, the log_2_ fold change relative to the starvation state is shown for both AA and Nitrate cultures across all time points (Before, Starvation, 1 d, 2 d, 3 d). AA-unique metabolites (e.g., (Z)-aconitic acid, 3-hydroxybutyric acid, γ-aminobutyric acid) already showed strong upregulation on day 1, which returned to baseline by day 3. Nitrate-unique metabolites (e.g., 3-hydroxyheptanoic acid, oxooctanoic acid, (±)-dihydrojasmonic acid) exhibited a delayed response, with upregulation appearing only on day 2–3. Common metabolites (e.g., NAD^+^, cyclic ADP-ribose, DL-glutamine) were upregulated in both sources but with higher amplitude and earlier onset in AA cultures. These patterns confirm that ammonium acetate triggers an immediate and broad metabolic reprogramming, whereas nitrate supports a slower, more restricted recovery.

Several metabolites were uniquely detected in AA cultures, pointing to active metabolic pathways associated with rapid nitrogen assimilation and cellular signalling. Cis-Aconitic acid ((Z)-aconitic acid) is a key intermediate of the tricarboxylic acid (TCA) cycle, formed from citrate by the enzyme aconitase [26]; its upregulation suggests enhanced TCA cycle activity and energy metabolism during early recovery. 3-Hydroxybutyric acid is a precursor of the biodegradable plastic polyhydroxybutyrate (PHB) and has been identified as a metabolite in heterotrophically grown *Chlorella* [27]; its accumulation may reflect a shift towards carbon storage or alternative energy metabolism. Notably, a recent study by Zhila et al. (2025) demonstrated that nitrogen limitation combined with acetate supplementation promotes polyhydroxyalkanoate accumulation in *Chlorella vulgaris* [28]. Biacetyl (2,3-butanedione) is a natural fermentation byproduct [29], though its specific role in microalgae remains unclear. γ-Aminobutyric acid (GABA) a well-established non-protein amino acid with a signalling role that regulates reactive oxygen species (ROS) homeostasis under environmental stress in microalgae [30,31]; in plants, stress-induced cytosolic Ca^2+^ activates Ca^2+^/calmodulin-dependent glutamate decarboxylase, leading to GABA synthesis [32]. Its presence indicates activation of stress signalling pathways during recovery. Tuberonic acid (12-hydroxyjasmonic acid) is a hydroxylated jasmonate that functions as a developmental signal and a metabolic shunt product in plants, mediating wound responses and defence pathways [33]; its detection suggests activation of jasmonate-related signalling in *N. semenenkoi*. Methylimidazoleacetic acid is a histamine metabolite involved in histidine metabolism [34], though its functional significance in algae is not well characterised. The sesquiterpene (2E,6E)-4-ethyl-7,11-dimethyl-2,6,10-dodecatrienal belongs to the terpenoid class and may participate in volatile organic compound (VOC)-mediated signalling or defence [35].

Conversely, metabolites unique to Nitrate cultures were enriched in fatty acid derivatives and intermediates of amino acid and nucleotide metabolism, possibly reflecting distinct metabolic adjustments under a nitrate-based nitrogen supply. 3-Hydroxyheptanoic acid is a medium-chain hydroxy fatty acid that can arise from β-oxidation and fatty acid metabolism [36,37,38], suggesting active fatty acid degradation. L-Seryl-L-serine is a dipeptide composed of two serine residues; serine is known to participate in amino acid transport and metabolism in *Chlorella* sp. [39,40]. Oxooctanoic acid is a medium-chain oxo-fatty acid identified in biological systems, including plants; its presence may indicate fatty acid oxidation [41]. Cyclo(His-Pro) is a cyclic dipeptide that has been detected in plants and shown to increase upon exposure to abiotic stress conditions and inhibit GAPC1 activity and modulate the NADPH/NADP^+^ ratio in *Arabidopsis* [42]; cyclic dipeptides are also known from marine algae [43]. N-Acetylglucosaminitol is related to N-acetylglucosamine, a key component of N-glycans in *Chlorella* microalgae [44]; its detection may reflect active glycoprotein or cell wall remodelling. (9E,11Z,13E,15Z)-4-Oxo-9,11,13,15-octadecatetraenoic acid is a polyunsaturated oxo-fatty acid found in fruits and seed oils [41], likely derived from lipid peroxidation or oxidation. 3-Ureidopropionic acid (N-carbamoyl-β-alanine) is an intermediate in uracil and pyrimidine metabolism [45]; its presence indicates active nucleotide turnover. Suberic acid is a dicarboxylic acid whose increased levels are attributed to lipid peroxidation under stress conditions in algae [46,47]. N-Acetyl-L-lysine (AC-LYS-OH) is an acetylated derivative of lysine involved in protein acetylation [48]. N-[(2S)-2-Hydroxypropanoyl]glycine is a glycine derivative, structurally related to aminophospholipids previously identified in brown algae [49].

Common metabolites upregulated in both sources included 1,3,6-trigalloyl glucose, a gallotannin polyphenolic compound found in various plants [50]; CMPF (3-carboxy-4-methyl-5-propyl-2-furanpropanoic acid), a furan fatty acid metabolite derived from polyunsaturated fatty acids, with a biosynthetic pathway proposed in algae [51]; and NAD^+^, a central redox cofactor essential for cellular energy metabolism and redox balance in *Chlorella* [52]. In our experiments, NAD^+^ showed similar dynamics in both acetate and nitrate media, which suggests its key role in maintaining energy and redox homeostasis of the cell, regardless of the nitrogen source. The restoration of NAD^+^ levels with the addition of nitrogen after starvation probably reflects the general need of cells to restore energy metabolism and redox balance to ensure active growth and biosynthetic processes. The similarity of the pattern in both variants indicates that NAD^+^ is one of the central metabolites reacting to nitrogen availability, and its dynamics does not depend on the nitrogen form, unlike many other metabolites, which differed significantly between ammonium and nitrate cultures.

Сyclic ADP-ribose (cADPR) is a metabolite of NAD^+^ that mobilises calcium from internal stores and links metabolism to cell cycle control in algae [53]. Cyclic ADP-ribose (cADPR) exhibited a similar pattern in both ammonium acetate and sodium nitrate cultures, with elevated levels during nitrogen starvation and a subsequent decrease after nitrogen replenishment, consistent with its role as a metabolic sensor linking cellular energy status to cell cycle regulation in microalgae. We also observed (Figure 6) that the amino acids arginine and glutamate are also the most affected substances and the patterns of their changes are very similar.

To further confirm that the observed ^15^N incorporation was not limited to amino acids, we examined the isotopic enrichment of several other nitrogen-containing metabolites that were reliably detected in the untargeted metabolomics data. Among nucleotides, guanine showed 50% enrichment on day 2 and reached 98.94% on day 3, while adenine exhibited 49.4% enrichment on day 2 and 100% on day 3. These values confirm that the purine nucleotide pool is actively synthesised de novo from the newly added ^15^N-labelled nitrogen source. For nicotinamide, a key precursor of the redox cofactor NAD^+^, enrichment reached 42.79% on day 2 and increased to 91.62% on day 3, indicating substantial turnover of the NAD^+^ pool during recovery. In contrast, the enrichment of choline was negligible (0–0.23%), consistent with its role as a structural component of membrane lipids rather than a rapidly turning-over nitrogen pool. Collectively, these examples demonstrate that the ^15^N label is incorporated into a broad range of nitrogen-containing compound classes beyond amino acids, supporting the conclusion that the recovery phase is characterised by widespread de novo biosynthesis.

## 3. Discussion

### 3.1. Similar Growth but Distinct Metabolic Recovery: Ammonium Versus Nitrate

The growth data demonstrated that *Neochlorella semenenkoi* IPPAS C-1210 is capable of efficient growth on both ammonium acetate and sodium nitrate, with virtually identical growth curves and final biomass yields. After nitrogen replenishment, all cultures resumed growth without any detectable lag, reaching dry biomass yields of ~2.8–3.0 g/L by day 10, regardless of the nitrogen source or isotopic label. This indicates that the strain is equally well adapted to utilize both ammonium and nitrate under the tested conditions, and that the energetic cost of nitrate reduction does not manifest as a detectable growth disadvantage within the resolution of our measurements. Previous studies on other *Chlorella* species have reported faster growth on ammonium [9,10] but our results show that *N. semenenkoi* does not exhibit such a preference under our experimental conditions. The lack of isotopic effects further confirms that ^15^N labeling is a reliable tool for metabolic tracing in this strain. It should be emphasized that part of the observed metabolic response is caused by the transition from nitrogen starvation to recovery. However, because both nitrogen sources were compared under identical starvation and recovery regimes, and at the same time points, the differences between AA and nitrate can be attributed specifically to the chemical form of nitrogen supplied. The starvation and recovery design therefore serves as a common background against which source-specific effects can be identified.

**Figure 7:**
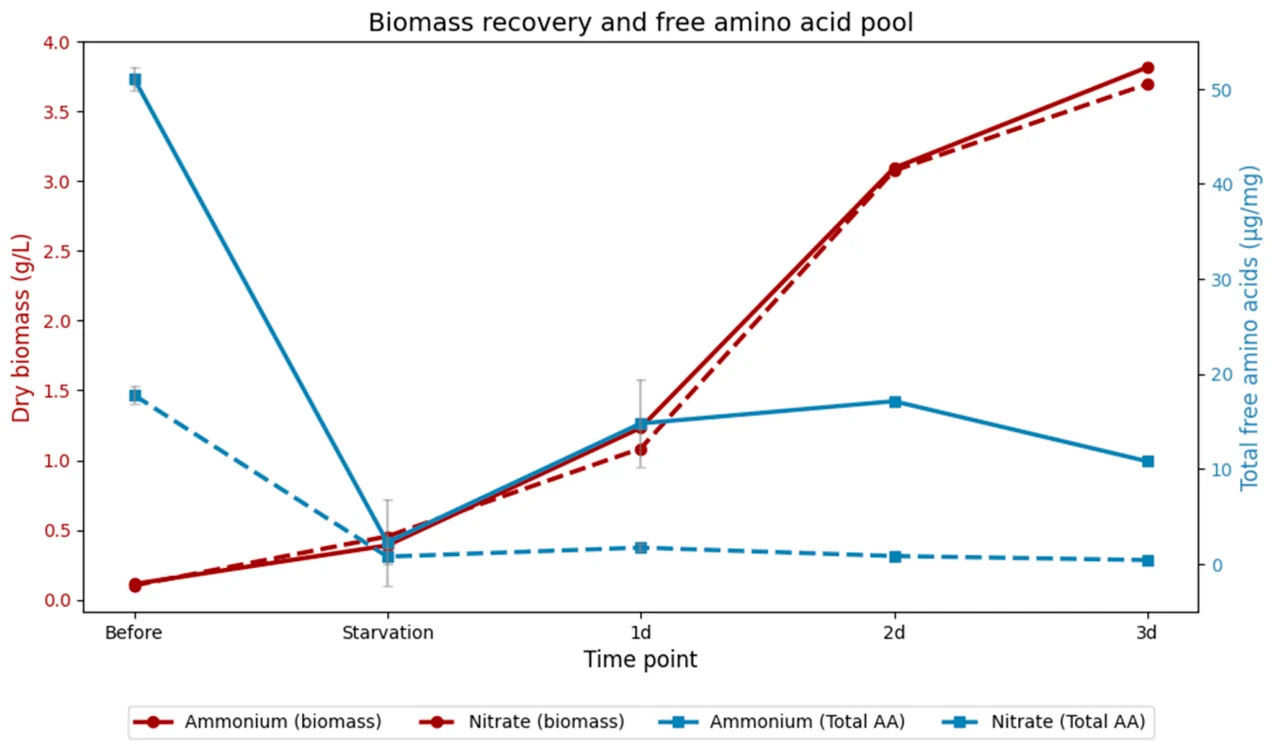
Relationship between biomass recovery and the total free amino acid pool during recovery from nitrogen starvation. Left axis (red): dry biomass (g/L). Right axis (blue): total free amino acid pool, calculated as the sum of all quantified amino acids (µg/mg lyophilized biomass). Solid lines, ammonium acetate; dashed lines, sodium nitrate. Note that Figure 2 shows individual amino acid concentrations, whereas Figure 7 shows the summed amino acid pool in relation to biomass accumulation; the two figures therefore address different questions and are not redundant.

However, despite the similar growth performance, the intracellular free amino acid pools differed markedly between the two nitrogen sources. To quantify this discrepancy, we calculated the total free amino acid pool (sum of all quantified amino acids, µg/mg biomass) and compared it with dry biomass accumulation (Figure 7). While dry biomass recovered to similar levels (~2.8–3.0 g/L) in both nitrogen sources by day 3, the total free amino acid pool in nitrate-grown cells (0.4 µg/mg) remained substantially lower than in ammonium-grown cells (15.5 μg/mg). Moreover, even in ammonium cultures, the pool did not reach the pre-starvation level (51.1 µg/mg). This indicates that the recovery of biomass is not accompanied by a proportional recovery of the free amino acid pool. The data suggest that nitrogen from nitrate, once assimilated, is rapidly channeled into proteins and other macromolecules rather than accumulating as free amino acids, resulting in a lower steady-state pool. This interpretation is supported by the near-complete ^15^N labeling of amino acids in both sources (Table 4), which confirms active de novo synthesis, and by the consistently lower free pool in nitrate cultures across all recovery days (Mann–Whitney U test, *p* < 0.05 for all days).

Comparable final biomass yields do not imply identical metabolic recovery. Biomass accumulation reflects the total macromolecular pool, whereas free amino acids represent a small dynamic fraction. Therefore, nitrate can support growth while maintaining a much lower free amino acid pool, likely by rapidly channelling newly assimilated nitrogen into proteins and other macromolecules.

The mechanistic basis for this difference lies in the energy requirements for nitrogen assimilation. Nitrate must first be reduced to nitrite and then to ammonium through the sequential action of nitrate reductase and nitrite reductase, consuming significant amounts of reducing equivalents [13]. In contrast, ammonium is already in a reduced state and can be directly incorporated into organic compounds via the glutamine synthetase/glutamate synthase (GS-GOGAT) cycle [15]. The additional energy demand for nitrate reduction likely results in a slower flux of nitrogen into the amino acid pool, which may be rapidly channeled into protein synthesis rather than accumulating as free amino acids. Similar patterns of differential amino acid accumulation have been reported for other microalgae [18].

Growth kinetics and final biomass yields were broadly similar between ^14^N and ^15^N cultures, although small differences in optical density were observed; because the ^15^N variant had *n* = 1, these differences cannot be formally assessed. The absence of a pronounced systematic deviation supports the use of ^15^N labelling for metabolic tracing while acknowledging the limitation of *n* = 1 for ^15^N [24,54].

### 3.2. Ammonium Acetate Drives Rapid Restoration of the Free Amino Acid Pool

The metabolomic analysis revealed that nitrogen starvation caused a pronounced depletion of most amino acids, consistent with the known role of amino acids as both nitrogen storage compounds and biosynthetic precursors during nutrient stress [3,4]. Upon nitrogen replenishment, the free amino acid pools recovered rapidly, but the kinetics and magnitude of recovery were strongly dependent on the nitrogen source.

**Figure 8:**
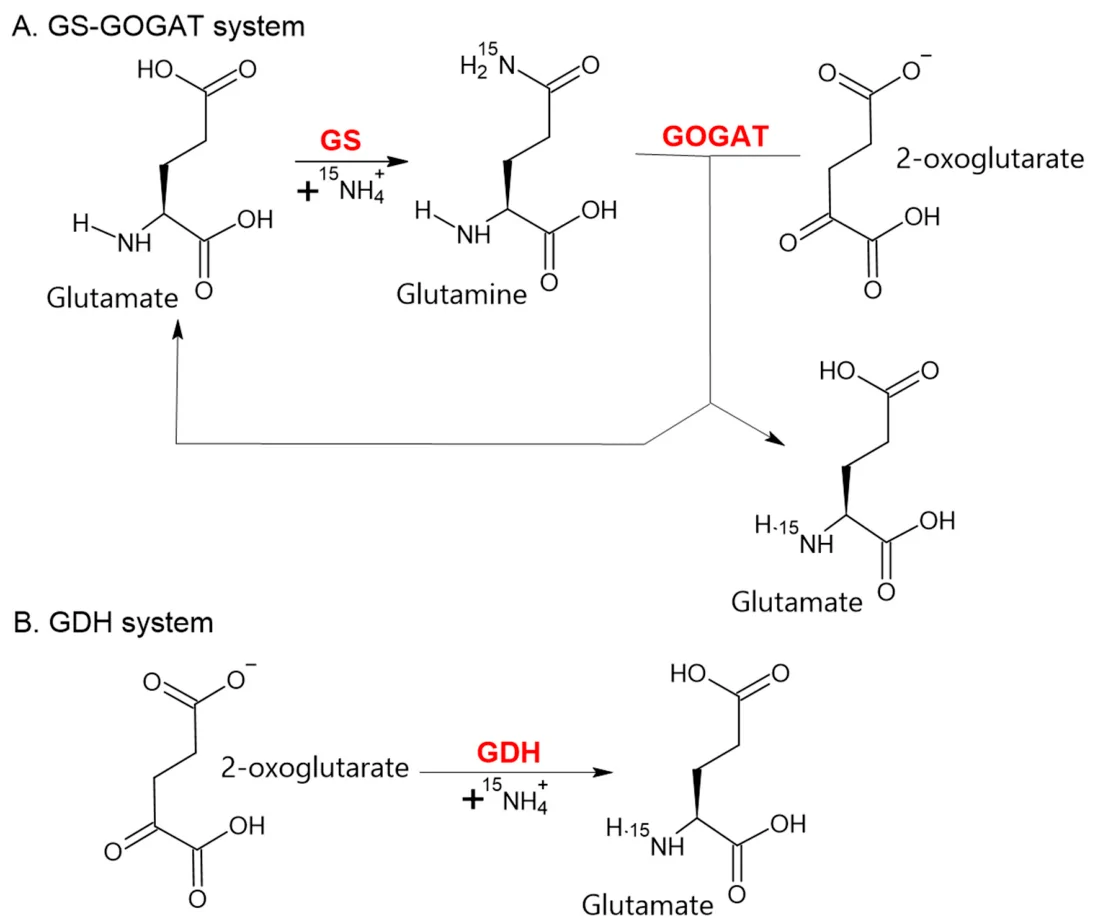
Metabolomic pathways of ammonium incorporation into amino acids in plant organisms. The first system includes glutamine synthase (GS) and glutamate synthase (glutamate-2-oxoglutarate aminotransferase, GOGAT), and the second includes glutamate dehydrogenase (GDH). Reproduced from our previous work on the same strain [22].

In ammonium acetate cultures, the majority of amino acids substantially recovered within 24 h of nitrogen addition, yet the total free amino acid pool reached only ~31% of the pre-starvation level on day 1, ~42% on day 2, and ~30% on day 3 (Figure 7). In nitrate cultures, the recovery was considerably more limited, reaching only ~15%, ~9%, and ~4.5% of the pre-starvation level on days 1, 2, and 3, respectively (Figure 7). Glutamate, the central hub of nitrogen assimilation, showed the most dramatic response, with concentrations reaching 3.31 µg/mg on day 1 (compared to 0.15 µg/mg in nitrate cultures; Table 1) and a log_2_(AA/Nitrate) ratio of 4.5, corresponding to a ~22-fold higher concentration. This pronounced accumulation of glutamate is consistent with its role as the primary acceptor of ammonium via the GS-GOGAT pathway [4,25] (Figure 8). The rapid incorporation of ammonium into glutamate and glutamine, followed by transamination to other amino acids, represents the core of primary nitrogen assimilation in microalgae [15].

The absence of several amino acids in nitrate cultures on days 2–3 means that their concentrations were below the limit of quantification. We cannot strictly distinguish true biological absence from analytical sensitivity limitations. However, the consistently low total amino acid pool and sustained growth suggest that these pools are extremely low rather than completely zero.

The especially high labeling in the glutamate/glutamine family (97–100% ^15^N enrichment) further supports the conclusion that these amino acids serve as the entry point for inorganic nitrogen into organic metabolism. Within 24 h of ^15^N-ammonium addition, essentially all glutamate and glutamine molecules were labeled, indicating complete replacement of pre-existing pools with newly synthesized molecules. This near-quantitative labeling demonstrates that the recovery phase is characterized by de novo biosynthesis rather than recycling of nitrogen from degraded proteins or other endogenous sources.

The initial burst of ammonium assimilation provides the building blocks for a broad spectrum of amino acids, but not all amino acids are synthesized simultaneously. Amino acids with a high nitrogen demand and rapid turnover (glutamate, glutamine, aspartate, asparagine, alanine) are synthesized first, whereas amino acids whose biosynthesis requires multiple enzymatic steps or specific carbon skeletons (proline, valine, leucine, isoleucine, phenylalanine) appear later or remain below the limit of quantification within the 3-day window. In addition, some amino acids may be rapidly consumed for protein synthesis and therefore do not accumulate in the free pool even when they are actively synthesized. This temporal hierarchy reflects the sequential nature of amino acid biosynthesis and the different metabolic fates of individual amino acids, rather than a selective absence of certain pathways.

### 3.3. Nitrate Supports Only Partial Recovery of the Free Amino Acid Pool

In contrast to ammonium acetate, nitrate cultures showed only a partial and delayed recovery of the free amino acid pool. Even after 3 days of recovery, the total free amino acid pool in nitrate-grown cells reached only ~4.5% of the pre-starvation level (Figure 7), and several amino acids (proline, valine, leucine, isoleucine, asparagine, phenylalanine) remained undetectable throughout the recovery period (Table 1, Figure 3A). For the amino acids that were detectable in both conditions (glutamate, glutamine, lysine, arginine, threonine, histidine), the log_2_(AA/Nitrate) ratios remained high (typically >1.5 on the log_2_ scale) throughout the recovery period, indicating that the differences between the two sources persisted rather than diminished; in several cases, the ratios even increased over time because the concentrations in AA cultures also declined towards the end of the recovery window, while in nitrate cultures they remained extremely low or undetectable (Figure 3A). Importantly, this delayed amino acid recovery occurred without any detectable lag in growth resumption (Figure 1), indicating that the cells were able to sustain biomass accumulation even when the free amino acid pool remained low.

The delayed recovery in nitrate cultures can be explained by the requirement for nitrate reduction before assimilation. The nitrate reductase enzyme system must be induced or activated upon nitrogen resupply, and the reducing power needed for nitrate reduction must be diverted from other metabolic processes [11,13]. Once nitrate is reduced to ammonium, however, the subsequent assimilation pathway is identical to that used for exogenous ammonium. This explains why the amino acid profiles eventually converge: both nitrogen sources feed into the same central nitrogen assimilation pathway, but ammonium provides a “jump start” by bypassing the reductive step.

We hypothesize that nitrate-derived nitrogen, after reduction to ammonium, is rapidly channelled into proteins and other macromolecules rather than accumulating in the free amino acid pool (Figure 7). Direct measurements of protein or macromolecular nitrogen pools were not performed in this study; therefore, this remains a hypothesis requiring validation by proteomic or ^15^N-protein tracing approaches. This interpretation is supported by the near-complete ^15^N labeling of amino acids in nitrate cultures (Table 4), which confirms active de novo synthesis, and by the fact that growth was not impaired despite the low free amino acid pool. Although ammonium has been shown to inhibit nitrate assimilation in *Chlorella vulgaris* when both sources are present simultaneously [11,12], in our study, the two nitrogen sources were supplied separately; the differences observed here therefore reflect the intrinsic kinetics of each assimilation pathway rather than direct inhibition.

The absence of several amino acids in nitrate cultures on days 2–3 means that their concentrations were below the limit of quantification. We cannot strictly distinguish true biological absence from analytical sensitivity limitations. However, the consistently low total amino acid pool and sustained growth suggest that these pools are extremely low rather than completely zero.

### 3.4. Near-Complete ^15^N Labeling Confirms De Novo Synthesis

The most striking finding of this study is the near-complete ^15^N enrichment of amino acids in both ammonium acetate and nitrate cultures. For ammonium acetate, the ^15^N enrichment exceeded 96% for all detectable amino acids as early as day 1 of recovery and remained stable throughout the subsequent days. For nitrate cultures, the enrichment was also 100% for the amino acids that were detectable (Lys, Glu, Met, His, Phe, Arg, Tyr, Trp), confirming that when amino acids are present, they are actively synthesized de novo from the added ^15^N-nitrate.

The high and stable labeling percentages indicate that the vast majority of amino acids present in the cells after nitrogen replenishment were synthesized from the newly added nitrogen source, rather than being recycled from pre-existing pools. This is consistent with the rapid depletion of amino acids during starvation (Table 1) and the subsequent rebuilding from fresh nitrogen. The stability of the labeling across days (standard deviations typically <2%) indicates that a steady state was reached rapidly and that the newly synthesized pools were continuously maintained.

The use of ^15^N labeling in this study also demonstrates the value of stable isotope tracing for understanding metabolic dynamics. While concentration measurements alone show the net change in pool sizes, isotopic labeling reveals the flux through biosynthetic pathways. The fact that ^15^N enrichment was high even for amino acids whose concentrations did not increase substantially (e.g., glutamate, which showed only a 6% concentration increase but 97% labeling) underscores the importance of flux analysis for understanding metabolic regulation. This observation aligns with our previous findings that the percentage of isotopic labeling differs markedly from quantitative concentration changes, making isotope labeling a more informative approach for studying metabolic dynamics than concentration measurements alone [22].

The high labeling in the glutamate/glutamine family (97–100%) is in full agreement with the central role of these amino acids in primary nitrogen assimilation via the glutamine synthetase/glutamate synthase (GS-GOGAT) pathway [4,25]. Notably, the percentage of labeling in glutamate (97%) was slightly lower than in glutamine (100%). This difference does not necessarily indicate a major contribution of glutamate dehydrogenase (GDH) to glutamate synthesis; rather, it reflects the fact that glutamate is a larger and more slowly turning-over pool compared to glutamine. Glutamine is synthesised exclusively through the GS pathway and is rapidly labelled, whereas glutamate participates in numerous metabolic reactions (transamination, catabolism, protein synthesis) and therefore contains a fraction of pre-existing, unlabeled molecules that have not yet been replaced within the 24-h labelling period. While GDH can function in glutamate synthesis in *Chlorella* under certain conditions [55], the high labelling of glutamine (100%) and the near-complete labelling of glutamate (97%) indicate that the GS-GOGAT pathway is the predominant route for ammonium assimilation in this strain. The proposed dominant role of the GS–GOGAT pathway is inferred from ^15^N-labelling patterns of glutamate and glutamine. Enzyme activities and gene expression were not measured in this study; therefore, this conclusion is mechanistically plausible but indirect.

The near-complete ^15^N enrichment should be interpreted as evidence that the detectable pool is dominated by de novo-synthesized molecules. For metabolites with very low pool sizes, enrichment values may be less reliable because signals approach the limit of detection. Such cases should therefore be considered indicative rather than strictly quantitative.

### 3.5. Broader Implications for Microalgal Biotechnology

The findings of this study have practical implications for the cultivation of *Neochlorella semenenkoi* for biotechnological applications. The ability of this strain to utilize both ammonium and nitrate, combined with its rapid metabolic recovery after nitrogen starvation, makes it a promising candidate for various cultivation strategies [56].

For applications requiring rapid restoration of the amino acid pool and enhanced production of nitrogen-rich metabolites—such as the production of protein-rich biomass or the recovery of nitrogen from wastewater—ammonium acetate appears to be the preferred nitrogen source. Although final biomass yields are similar for both sources, ammonium enables a much faster and more complete recovery of the free amino acid pool, which is essential for protein synthesis and cell growth. The immediate resumption of metabolic activity upon ammonium addition also suggests that this strain could be effectively used in fed-batch or continuous cultivation systems where nitrogen pulses are used to control growth and product formation.

For applications where nitrate is the more available or cost-effective nitrogen source (e.g., in certain wastewater streams or agricultural runoff), the strain can still achieve comparable biomass yields. However, the amino acid profiles do not fully converge; only a subset of amino acids (glutamate, glutamine, lysine, arginine, threonine, histidine) recovers to levels comparable to those in ammonium cultures, while many others (proline, valine, leucine, isoleucine, asparagine, phenylalanine) remain undetectable even after 3 days. Thus, nitrate can sustain growth but does not fully restore the metabolic profile observed with ammonium. This makes nitrate a viable alternative when ammonium is unavailable, but with a trade-off in the speed and completeness of metabolic recovery.

The absence of an isotopic effect on growth and metabolism also validates the use of ^15^N labeling for metabolic flux analysis in this strain. This opens the door for more detailed flux studies that could quantify the rates of nitrogen assimilation, transamination, and amino acid catabolism under different growth conditions. Such studies would provide deeper insights into the regulatory mechanisms that govern nitrogen metabolism in this promising microalga.

However, it should be noted that these conclusions are limited to laboratory-scale experiments with a single strain under controlled pH, light, and CO_2_ conditions. Scale-up will require consideration of salt costs, pH control, acetate accumulation, oxygen transfer, contamination risk, and overall process economics.

An important caveat of this study is that ammonium acetate provides not only nitrogen but also acetate, which can serve as an additional organic carbon source. Acetate can be taken up and metabolized by many microalgae, including Chlorella-like species, and may contribute to the observed metabolic differences independently of the nitrogen form. In particular, the early upregulation of TCA cycle intermediates and the accumulation of 3-hydroxybutyric acid in ammonium-acetate cultures could partly reflect acetate metabolism rather than ammonium assimilation. Therefore, the comparison between ammonium acetate and sodium nitrate should be interpreted as a comparison of two cultivation regimes that differ in both the nitrogen form and the availability of organic carbon, rather than as a strictly isolated effect of the nitrogen source. Future studies using ammonium chloride or ammonium sulfate as the ammonium source, without acetate, would help to disentangle these effects.

### 3.6. Untargeted Metabolomics Reveals Global Metabolic Reprogramming During Recovery

The untargeted metabolomics analysis expanded our understanding beyond amino acids, revealing a profound and coordinated reprogramming of nitrogen-rich metabolites, signaling molecules, and membrane lipids during recovery. Comparison of metabolite profiles between AA and nitrate cultures showed that AA supported a broader metabolic repertoire, with 250 detectable metabolites versus 132 in nitrate, and a larger number of unique metabolites (164 vs. 46; Table 6). This reflects the more direct role of ammonium in nitrogen assimilation and the production of nitrogen-rich secondary metabolites.

The heatmaps of the most variable metabolites (Figure 6) revealed distinct temporal patterns depending on the nitrogen source and metabolite category. In AA cultures, the majority of metabolites already showed early and robust upregulation on day 1, with a gradual return to baseline by day 3. This pattern was particularly pronounced for TCA cycle intermediates (e.g., cis-aconitic acid), stress-signalling molecules (GABA, tuberonic acid, pyrone), and precursors of cell wall and nucleotide metabolism. The simultaneous upregulation of these metabolites indicates that ammonium triggers a coordinated activation of central carbon metabolism, stress signalling, and biosynthetic pathways during early recovery.

In contrast, nitrate-unique metabolites exhibited a delayed response, with upregulation appearing only on day 2–3. These metabolites were enriched in amino acid and nucleotide derivatives and stress-related compounds. Importantly, many of the fatty-acid-derived metabolites in this group were already elevated during starvation and remained high in nitrate cultures throughout the recovery period, suggesting that nitrate-grown cells rely more on lipid turnover and oxidative stress metabolism rather than on immediate activation of biosynthetic pathways. This pattern may reflect a slower activation of central carbon metabolism due to the energy cost of nitrate reduction, and a greater reliance on internal carbon and nitrogen recycling. Direct measurements of ROS, MDA/TBARS, or antioxidant enzyme activities were not performed; therefore, the oxidative-stress interpretation remains hypothetical and requires dedicated experiments.

Common metabolites upregulated in both sources included the redox cofactor NAD^+^ and its downstream signalling molecule cyclic ADP-ribose (cADPR), as well as several amino acids and amino acid derivatives (DL-arginine, DL-glutamine, L-(−)-asparagine, methionylserine). The polyphenol 1,3,6-trigalloyl glucose was strongly suppressed during recovery in both sources, while the furan fatty acid CMPF and 7-hydroxyoctanoic acid showed source-dependent dynamics. NAD^+^ and cADPR showed similar dynamics in AA and nitrate cultures, consistent with their roles as metabolic sensors linking cellular energy status to cell cycle regulation in microalgae [53]. The similarity of this pattern in both sources underscores that these molecules are fundamental regulators of the metabolic response to nitrogen availability.

The heatmaps also revealed a shift from storage and stress responses towards active growth. In the “Common” panel, 1,3,6-trigalloyl glucose, a polyphenol typically associated with stress and storage, was markedly suppressed during recovery in both sources. Conversely, the amino acids DL-arginine and DL-glutamine—key nitrogen carriers—were strongly upregulated, with a higher amplitude and earlier onset in AA cultures than in nitrate cultures. Together with the upregulation of NAD^+^ and cADPR, this pattern reflects activation of central nitrogen metabolism and redox-related signalling during recovery.

Importantly, the ^15^N isotopic enrichment extended beyond amino acids to other nitrogen-containing metabolites. Nucleotides (guanine, adenine) and the NAD^+^ precursor nicotinamide showed substantial enrichment by day 2–3 (up to 98–100%), confirming active de novo synthesis of purine nucleotides and the NAD^+^ pool from the newly added ^15^N-labelled nitrogen source. In contrast, choline showed negligible enrichment, consistent with its role as a structural membrane component rather than a rapidly turning-over nitrogen pool. These data demonstrate that the ^15^N label is incorporated into a broad range of nitrogen-containing compound classes, supporting the conclusion that the recovery phase is characterised by widespread de novo biosynthesis.

Together, these data show that ammonium acetate triggers an immediate, broad, and coordinated metabolic reprogramming involving central carbon metabolism, stress signalling, and redox-related signalling, whereas nitrate supports a slower, more restricted recovery that relies more on lipid catabolism and oxidative stress responses. This global view, combined with the targeted amino acid analysis, provides a comprehensive picture of how nitrogen source availability shapes the metabolic landscape of *N. semenenkoi* during recovery from starvation.

## 4. Materials and Methods

### 4.1. Microalgae Strain and Cultivation Conditions

The axenic green alga *Neochlorella semenenkoi* IPPAS C-1210 was obtained from the Collection of Microalgae and Cyanobacteria IPPAS at the K.A. Timiryazev Institute of Plant Physiology, Russian Academy of Sciences (Moscow, Russia). Before the experiment, the strain was cultivated under sterile conditions for 10–14 days in 300-mL Erlenmeyer flasks filled with 100 mL of ½ Tamiya modified medium [57] at 27 °C under continuous luminescent illumination of 50 μmol photons m^−2^ s^−1^ in an MLR-352-PE growth chamber (Panasonic, Japan).

In the present study, two variants of BG-11 medium were used. The first was standard BG-11 medium containing sodium nitrate (NaNO_3_, Nit) as the nitrogen source [58] while the second was modified by replacing NaNO_3_ with ammonium acetate (CH_3_COONH_4_, NH_4_Ac, or AA) at an equimolar nitrogen concentration of 17.6 mM. Both media were supplemented with 20 mM HEPES (pH 7.5) to maintain a constant pH during intensive growth, thereby preventing alkalization caused by photosynthesis and acidification resulting from ammonium consumption.

Before the main experiment, pre-cultivation was carried out for four days in 250 mL of sterile buffered BG-11 medium containing the respective nitrogen source (either Nit or AA) using a laboratory system for intensive cultivation [59]. The cultures were grown at constant temperature of 32 ± 1 °C under continuous bilateral LED illumination at a combined intensity of 600 μmol photons m^−2^ s^−1^ and with sparging of a sterile gas-air mixture containing 1.3–1.8% CO_2_ [59].

After four days of growth, cultures reached a dry biomass concentration of ~3 g/L and were used as inoculum for the main experiment. Cells from each medium were harvested by centrifugation, washed twice with nitrogen-free BG-11 medium (BG-11–N), and resuspended in two fresh vessels with 250 mL of BG-11–N to induce nitrogen starvation. Initial biomass concentration was adjusted to ~0.1 g/L in all four vessels. Nitrogen starvation was then carried out for three days under the same cultivation conditions.

After the starvation period, the cultures were replenished with their respective nitrogen sources. For each nitrogen source (nitrate or ammonium acetate), the experiment was performed in two parallel sets: one set received the unlabeled (^14^N) form, and the other received the isotopically labeled (^15^N) form of the same nitrogen compound (Na^15^NO_3_ or ^15^NH_4_OAc). The final nitrogen concentration after replenishment was 17.6 mM. The required amount of each nitrogen source was dissolved in 1 mL of deionized water, filter-sterilized, and added to the cultures.

Samples for metabolomic analysis were collected at three time points:

1. Before starvation—after 4 days of growth on full medium with the respective nitrogen source (day 0 of the starvation phase);
2. Under nitrogen starvation—after 3 days of incubation in nitrogen-free medium (day 3 of starvation);
3. Recovery phase—at 1, 2, and 3 days after the addition of the nitrogen source (either ^14^N- or ^15^N-labelled).

At each time point, cells were harvested by centrifugation, washed with distilled water, frozen at –80 °C, lyophilized, and kept in darkness at ambient temperature until further analysis.

Growth was monitored by measuring optical density and dry weight. Optical density was measured at 680 and 750 nm using a Genesys 10S UV-Vis spectrophotometer (Thermo Scientific, Waltham, MA, USA). For dry weight determination, aliquots of 1–2 mL were collected by centrifugation, washed with distilled water, and dried at 80 °C for 12 h in pre-weighed plastic microtubes in a drying oven. Dry weights were determined in triplicate.

For each nitrogen source (NaNO_3_ or NH_4_Ac), the ^14^N-labelled recovery variant was performed in three parallel cultivation vessels (n = 3 biological replicates), whereas the ^15^N-labelled variant was performed in one vessel (n = 1) because of the high cost of isotopically labelled salts. Therefore, all concentration values for ^14^N in tables and figures represent mean values across three biological replicates; values for ^15^N are single determinations. Dry weight was measured in triplicate. HPLC-MS technical injections were used for analytical quality control, but biological replication for ^14^N was used for statistical comparisons; no formal statistical testing was performed for ^15^N due to *n* = 1.

### 4.2. Sample Preparation for Polar Metabolites

Lyophilized algal biomass (10 mg) was extracted using a modified Folch liquid–liquid extraction procedure [4]. Briefly, 200 µL of ultrapure water and 320 µL of cold methanol were added to the biomass, and the mixture was ultrasonicated for 1 h to ensure complete homogenization. Then, 640 µL of cold chloroform was added, and the sample was incubated for 20 min at 4 °C in a thermoshaker with constant agitation. Subsequently, 300 µL of ultrapure water was added, and incubation continued for another 10 min at 4 °C. The tubes were centrifuged at 2000× *g* for 5 min at 4 °C to achieve phase separation.

After centrifugation, the upper (aqueous) phase, containing polar metabolites, was carefully transferred to a new tube. The lower (organic) phase, containing total lipids, was collected separately; lipid analysis was not performed in this study. To maximize recovery of polar metabolites, the remaining pellet and interphase were re-extracted with 250 µL of chloroform:methanol (2:1, *v*/*v*), centrifuged again, and the upper phase was combined with the first aqueous extract. The combined polar fractions were dried in a vacuum concentrator without heating and reconstituted in 200 µL of 80% acetonitrile. The resulting extracts were stored at –20 °C prior to HPLC-MS analysis.

### 4.3. Preparation of Calibration Standards for Amino Acid Quantification

For semi-quantitative analysis of amino acids, analytical grade standards were obtained from commercial suppliers: alanine (Sigma), arginine (CDH Productions), asparagine (Panreac), aspartic acid (CDH Productions), cysteine (Sigma), glutamic acid (CDH), glutamine (Panreac), glycine (Sigma), histidine (CDH Productions), isoleucine (Sigma), leucine (Sigma), lysine (CDH Productions), phenylalanine (CDH Productions), proline (CDH Productions), serine (Sigma), threonine (CDH Productions), tryptophan (CDH Productions), and tyrosine (CDH Productions).

Each amino acid standard (5 mg) was dissolved in 2 mL of ultrapure water to prepare a stock solution. A series of calibration standards was then prepared by sequential dilution of the stock solution to final concentrations of 90, 45, 9, 4.5, 0.9, 0.45, 0.09, 0.045, and 0.009 µg/mL. Aliquots of 250 µL of each calibration standard were dried in a vacuum concentrator without heating and reconstituted in 80% acetonitrile, following the same protocol used for the biological samples. For amino acids, quantification was performed using external calibration curves. The calibration curves were constructed by plotting peak area versus concentration for each amino acid (Supplementary Figures S1–S17). The concentrations in the samples were calculated using these calibrations. Values below the are indicated as <LOQ or ND and were excluded from calculations of medians, interquartile ranges, and statistical tests. When calculating AA/nitrate ratios, if a metabolite was not detected in nitrate, the ratio was not calculated; in the heatmap such cases are marked as 0.00 and explained in the legend. Biologically, these cases are interpreted as ‘below the limit of detection’, not as absolute absence.

### 4.4. HPLC-MS/MS Analysis

HPLC-MS/MS analysis was performed on an LTQ Orbitrap Velos mass spectrometer (Thermo Scientific Inc., Waltham, MA, USA) coupled with a Thermo Scientific Accela 600 HPLC system. Chromatographic separation was carried out on a Waters Acquity UPLC BEH HILIC column (2.1 × 150 mm, 1.7 µm particle size) using the following gradient elution with mobile phase A (0.1% formic acid and 0.176% ammonium formate in water) and mobile phase B (0.1% formic acid and 0.176% ammonium formate in acetonitrile): 0–1 min, 100% B; 1–20 min, 100% to 10% B; 20–22 min, 10% B; 22–25 min, 10% to 100% B; 25–33 min, 100% B. The flow rate was maintained at 0.2 mL/min, and the column temperature was set at 20 °C.

Mass spectrometry was performed in both positive and negative electrospray ionization (ESI) modes with spray voltages of 4.5 kV and 3.5 kV, respectively. Data acquisition was carried out in full-scan mode at a resolution of 60,000 (m/z 400), followed by data-dependent MS/MS (Top5) with stepped collision energy (10, 30, and 45 V). The isolation window was set to 1 Da. For each sample, 5 µL of extract was injected.

### 4.5. Data Processing and Metabolite Identification

Raw HPLC-MS data were processed using XCalibur 4.0 (Thermo Scientific), Compound Discoverer 3.3 (Thermo Scientific), and MS-DIAL 4.20 software [60,61]. The processing workflow included peak detection, deconvolution, deisotoping, and alignment across samples. For metabolite annotation, accurate masses and MS/MS fragmentation spectra were matched against the mzCloud and MassBank databases [62] using Compound Discoverer and MS-DIAL with a mass accuracy tolerance of 10 ppm and an annotation score threshold of 70%. Amino acids were confirmed using authentic standards and external calibration curves (Supplementary Figures S1–S17). For other metabolites, annotation confidence corresponds to MSI Level 2–3. A full annotation table including m/z, retention time, adduct, database, score, and MSI level is provided in Supplementary Tables S2 and S3. For untargeted metabolomics, features with a coefficient of variation < 30% across technical replicates were retained for further analysis.

^15^N-enrichment percentages were calculated directly from the extracted ion chromatograms of the ^14^N- and ^15^N-containing isotopologues of each detected metabolite (∆M(^15^N − ^14^N) = 0.997). For amino acids, the percentage of ^15^N labeling was determined as the ratio of the ^15^N-isotopologue peak area to the sum of the ^14^N- and ^15^N-isotopologue peak areas, multiplied by 100.

Statistical comparisons between ammonium acetate and nitrate cultures were performed using only ^14^N data. For each recovery day, all detected amino acids were pooled and compared between nitrogen sources using the non-parametric Mann–Whitney U test (n = 17 amino acids per condition, as indicated in Table 2). Normality was assessed using the Shapiro–Wilk test where appropriate. Multiple comparisons across recovery days were corrected using the Benjamini–Hochberg false discovery rate (FDR) procedure. Formal statistical testing between ^14^N and ^15^N cultures was not performed because the ^15^N variant had *n* = 1; these comparisons are presented descriptively. A *p*-value < 0.05 was considered significant. All calculations were performed in Python 3.13 (SciPy library). Values marked as <LOQ (peak detected but below the lowest calibration standard) and ND (no peak detected) were excluded from quantitative calculations and statistical tests. They were not treated as zero. In the pooled analysis, the unit of observation is the individual amino acid (n = 17 detected amino acids), not the biological replicate. This analysis therefore compares the distribution of concentrations across amino acids between nitrogen sources, rather than testing differences at the level of individual biological replicates. This approach is used as a summary measure of the overall amino acid pool, and it does not replace metabolite-specific analysis, which is presented in Table 1 and Figure 2B, Figure 3 and Figure 4.

## 5. Conclusions

This study provides a comprehensive comparative analysis of nitrogen assimilation and metabolic recovery in *Neochlorella semenenkoi* IPPAS C-1210 under two inorganic nitrogen sources. The strain efficiently utilizes both ammonium acetate and sodium nitrate, showing comparable growth kinetics and final biomass yields (~2.8–3.0 g/L), and the absence of an isotopic effect validates the use of ^15^N labelling for metabolic tracing. Ammonium acetate acts as a “jump start” source, enabling rapid and broad restoration of the free amino acid pool dominated by the glutamate/glutamine hub, whereas nitrate supports a slower and only partial recovery, with several amino acids remaining below the limit of quantification even after three days. Normalization to biomass confirmed that nitrate-grown cells contain substantially fewer free amino acids per unit of biomass, which we interpret as rapid channelling of assimilated nitrogen into macromolecules rather than accumulation in the free pool; this remains a hypothesis, as macromolecular nitrogen pools were not measured directly.

Untargeted metabolomics revealed early upregulation of TCA cycle intermediates, stress-signalling molecules, and nucleotide precursors in ammonium cultures, versus a delayed, lipid-centric response in nitrate cultures, with the oxidative-stress component remaining hypothetical because ROS and lipid peroxidation were not measured directly; NAD+ and cyclic ADP-ribose showed conserved dynamics across both sources.

^15^N tracing confirmed that recovery is driven mainly by de novo biosynthesis in the detectable pools, with near-complete labelling (>96%) of amino acids and enrichment extending to nucleotides and the NAD^+^ precursor nicotinamide, and the high labelling of the glutamate/glutamine family (97–100%) supports a dominant role of the GS–GOGAT pathway, although this is inferred from labelling patterns rather than from direct enzyme or gene expression measurements.

Under the laboratory conditions tested here and for this strain, ammonium acetate may be preferable for applications requiring rapid metabolic recovery and enhanced production of nitrogen-rich compounds, while nitrate remains a viable alternative with a trade-off in recovery kinetics; scale-up will require consideration of salt costs, pH control, acetate accumulation, oxygen transfer, contamination risk, and overall process economics.

Our findings have direct biotechnological implications: under the laboratory conditions tested here and for this strain, ammonium acetate may be preferable for applications requiring rapid metabolic recovery and enhanced production of nitrogen-rich compounds (e.g., protein-rich biomass, secondary metabolites), while nitrate remains a viable alternative when ammonium is unavailable or cost-prohibitive, albeit with a trade-off in the speed and completeness of metabolic recovery.

This work lays the groundwork for optimising large-scale cultivation protocols and for developing metabolic engineering strategies to enhance the production of high-value compounds in *N. semenenkoi*. The combination of targeted amino acid quantification, untargeted metabolomics, and ^15^N isotopic tracing provides a powerful framework for understanding nitrogen metabolism in photosynthetic microorganisms and can be extended to other industrially relevant strains. The main limitations of this study are the single biological replicate for ^15^N-labelled cultures, the absence of direct protein/macromolecular nitrogen measurements, and the putative nature of most metabolite annotations.

## Figures and Tables

**Table 1:** Intracellular mean concentrations of amino acids (µg per mg of lyophilized biomass) in *Neochlorella semenenkoi* IPPAS C-1210 under two nitrogen sources: ammonium acetate (AA) and sodium nitrate (Nit). Cells were sampled before starvation (Before), after 3 days of nitrogen starvation (Starvation), and at 1, 2, and 3 days after replenishment with either an unlabeled (^14^N) or isotopically labeled (^15^N) nitrogen source. Values were calculated from HPLC-MS/MS peak areas using external calibration curves. Abbreviations: AA, ammonium acetate; Nit, sodium nitrate; <LOD, below the limit of quantification (peak detected but below the lowest calibration standard). ND—not detected (no peak). For ^14^N, values are means of three biological replicates (*n* = 3). For ^15^N, values are single determinations (n = 1).

Amino Acid	Before		Starvation		+^14^N, 1 Day		+^14^N, 2 Day		+^14^N, 3 Day		+^15^N, 1 Day		+^15^N, 2 Day		+^15^N, 3 Day	
	**AA**	**Nit**	**AA**	**Nit**	**AA**	**Nit**	**AA**	**Nit**	**AA**	**Nit**	**AA**	**Nit**	**AA**	**Nit**	**AA**	**Nit**
Alanine	3.548	ND	ND	ND	2.866	ND	1.258	ND	ND	ND	1.209	ND	ND	ND	ND	ND
Arginine	7.908	4.007	<LOD	<LOD	3.858	2.178	2.132	0.727	4.112	0.473	4.264	0.636	2.527	<LOD	1.375	ND
Asparagine	0.959	0.168	ND	ND	0.438	<LOD	1.019	<LOD	0.276	ND	0.519	ND	0.097	ND	<LOD	ND
Aspartic acid	3.331	0.541	ND	ND	0.436	ND	1.508	ND	2.054	ND	0.916	ND	1.082	ND	0.387	ND
Glutamic acid	20.163	11.315	1.942	0.443	3.309	0.146	11.570	0.778	5.672	0.252	2.444	<LOD	7.060	<LOD	3.478	ND
Glutamine	0.437	0.118	0.027	ND	0.243	0.030	0.693	0.034	0.531	ND	0.387	ND	0.235	ND	0.079	ND
Histidine	0.120	0.066	0.031	0.021	0.051	0.026	0.028	ND	0.056	ND	0.060	ND	0.069	ND	0.070	ND
Isoleucine	0.282	<LOD	<LOD	ND	0.020	<LOD	0.007	ND	0.054	ND	0.036	ND	<LOD	ND	<LOD	ND
Leucine	0.188	<LOD	<LOD	ND	0.212	<LOD	0.019	ND	<LOD	ND	0.225	ND	<LOD	ND	<LOD	ND
Lysine	5.023	1.242	0.268	0.296	1.035	0.251	0.437	0.110	0.673	0.072	1.210	0.066	0.632	ND	0.360	ND
Phenylalanine	0.191	<LOD	<LOD	<LOD	0.157	<LOD	0.121	<LOD	0.087	<LOD	0.178	<LOD	0.053	ND	0.062	ND
Proline	1.622	<LOD	ND	<LOD	1.112	<LOD	0.767	<LOD	0.664	<LOD	0.557	ND	0.164	ND	0.054	ND
Serine	3.305	0.116	ND	ND	0.994	ND	1.124	ND	0.541	ND	0.590	ND	0.315	ND	0.099	ND
Threonine	1.932	0.193	ND	ND	0.746	0.082	0.649	ND	0.548	ND	0.628	ND	0.325	ND	0.159	ND
Tryptophan	<LOD	<LOD	<LOD	<LOD	<LOD	<LOD	<LOD	<LOD	<LOD	<LOD	<LOD	<LOD	<LOD	ND	ND	ND
Tyrosine	0.973	<LOD	<LOD	ND	<LOD	<LOD	0.094	<LOD	0.100	<LOD	0.114	<LOD	0.018	ND	<LOD	ND
Valine	1.105	<LOD	<LOD	ND	0.484	<LOD	0.237	ND	0.098	ND	0.256	ND	<LOD	ND	<LOD	ND
Alanine	3.548	ND	ND	ND	2.866	ND	1.258	ND	ND	ND	1.209	ND	ND	ND	ND	ND
Arginine	7.908	4.007	<LOD	<LOD	3.858	2.178	2.132	0.727	4.112	0.473	4.264	0.636	2.527	<LOD	1.375	ND

**Table 2:** Statistical comparison (Mann–Whitney U test) of total amino acid concentrations between ammonium acetate (AA) and sodium nitrate (Nitrate) cultures on each day of recovery (only ^14^N data). Values are median ± interquartile range (IQR) across all detected amino acids (Table 1). *p*-values were adjusted for multiple comparisons using the Benjamini–Hochberg FDR procedure. *p*-values were adjusted for multiple comparisons using the Benjamini–Hochberg false discovery rate (FDR) procedure. Significant differences (*p* < 0.05) are indicated in bold.

Day	AA (µg/mg), Median ± IQR	Nitrate (µg/mg), Median ± IQR	U-Statistic	Raw *p*-Value	FDR-Adjusted *p*-Value
**1 d**	0.63 ± 1.21	0.00 ± 0.22	168.00	**0.0047**	**0.0141**
**2 d**	0.49 ± 0.86	0.00 ± 0.16	115.50	**0.0142**	**0.0159**
**3 d**	0.42 ± 0.72	0.00 ± 0.13	87.00	**0.0159**	**0.0159**

**Table 3:** Descriptive comparison of total amino acid concentrations between ^14^N and ^15^N cultures for each nitrogen source and recovery day. Formal statistical testing was not performed because ^15^N had *n* = 1. ^14^N data are sums of all quantified amino acids from three biological replicates (n = 3); values are therefore means. ^15^N data are single determinations (n = 1). Abbreviations: AA, ammonium acetate; ND, not determined (most amino acids below the limit of quantification).

Source	Day	^14^N Total Amino Acids, µg/mg (Mean, *n* = 3)	^15^N Total Amino Acids, µg/mg (Single Value, *n* = 1)
**AA**	1 d	15.96	13.59
**AA**	2d	21.66	12.56
**AA**	3 d	15.47	6.12
**Nitrate**	1 d	2.71	0.70
**Nitrate**	2 d	1.65	ND
**Nitrate**	3 d	0.80	ND

**Table 4:** ^15^N-enrichment percentages for amino acids in *Neochlorella semenenkoi* IPPAS C-1210 during recovery from nitrogen starvation. Values represent the percentage of ^15^N-containing molecules in the total pool for each amino acid at 1, 2, and 3 days after nitrogen replenishment with ^15^N-ammonium acetate. Mean and standard deviation (std) across the three time points are also provided. Abbreviations: n.d., not detected (below the limit of quantification). *—the amino acid was found in only one sample.

Amino Acid	1 Day	2 Day	3 Day	Mean ± SD	1 day	2 Day	3 Day	Mean ± SD
	**Ammonium Acetate**				**Sodium Nitrate**			
**Alanine**	100.0	97.0	97.0	98.0 ± 1.7	n.d.	n.d.	n.d.	–
**Arginine**	98.0	97.0	97.0	97.3 ± 0.6	98.0	n.d.	n.d.	98.0 ± 0.0 *
**Asparagine**	100.0	100.0	100.0	100.0 ± 0.0	n.d.	n.d.	n.d.	–
**Aspartic acid**	100.0	100.0	100.0	100.0 ± 0.0	n.d.	n.d.	n.d.	–
**Glutamic acid**	97.0	97.0	97.0	97.0 ± 0.0	100.0	100.0	100.0	100.0 ± 0.0
**Glutamine**	100.0	100.0	100.0	100.0 ± 0.0	n.d.	n.d.	n.d.	–
**Histidine**	100.0	100.0	100.0	100.0 ± 0.0	100.0	n.d.	n.d.	100.0 ± 0.0 *
**Isoleucine**	98.0	97.0	97.0	97.3 ± 0.6	n.d.	n.d.	n.d.	–
**Leucine**	98.0	97.0	97.0	97.3 ± 0.6	n.d.	n.d.	n.d.	–
**Lysine**	100.0	100.0	100.0	100.0 ± 0.0	100.0	100.0	n.d.	100.0 ± 0.0 *
**Methionine**	n.d.	n.d.	n.d.	–	100.0	100.0	100.0	100.0 ± 0.0
**Phenylalanine**	96.0	97.0	96.0	96.3 ± 0.6	100.0	n.d.	n.d.	100.0 ± 0.0 *
**Proline**	98.0	97.0	97.0	97.3 ± 0.6	n.d.	n.d.	n.d.	–
**Serine**	100.0	100.0	100.0	100.0 ± 0.0	n.d.	n.d.	n.d.	–
**Threonine**	99.0	97.0	97.0	97.7 ± 1.2	n.d.	n.d.	n.d.	–
**Tryptophan**	100.0	100.0	100.0	100.0 ± 0.0	100.0	n.d.	n.d.	100.0 ± 0.0 *
**Valine**	100.0	96.0	85.0	93.7 ± 7.8	n.d.	n.d.	n.d.	–
**Tyrosine**	100.0	100.0	100.0	100.0 ± 0.0	100.0	n.d.	n.d.	100.0 ± 0.0 *

**Table 5:** Classification of identified metabolites in *Neochlorella semenenkoi* IPPAS C-1210 under the experimental conditions.

Class	Count	Representative Compounds
**Amino acids and derivatives**	60	L-Arginine, L-Aspartic acid, L-Serine, 4-Oxoproline, L-Tyrosine, 3-Hydroxy-L-proline, DL-Glutamine, Methionylserine, O-Acetylserine, DL-Tryptophan, L-Histidine, L-Lysine, L-Glutamic acid, L-(−)-Threonine, L-Leucine, L-(+)-Asparagine, Betaine, Choline
**Organic acids**	57	Citric acid, Succinic acid, L-(−)-Malic acid, 3-Hydroxybutyric acid, Azelaic acid, Propionic acid, Maleic acid, Mevalonic acid, 2-Aminoadipic acid, Methylmalonic acid, Hippuric acid, Ethylmalonic acid, (Z)-Aconitic acid
**Lipids and fatty acids**	33	(+/−)13-HODE, 9(S)-HpOTrE, 13(S)-HOTrE, Corchorifatty acid F, (+/−)9,10-dihydroxy-12Z-octadecenoic acid, 9,10-Dihydroxystearic acid, Dihydrolipoic acid, 1-Monolinolein, 2,3-Dinor-11β-prostaglandin F2α, Ubiquinol, Palmitic Acid, Ricinoleic Acid, Stearidonic acid
**Nucleotides and bases**	26	5′-S-Methyl-5′-thioadenosine, Adenine, Adenosine, Cytidine, Cytosine, Guanine, Hypoxanthine, Uridine, Xanthine, Xanthosine, 1-Methylxanthine, Cyclic ADP-ribose, UDP-N-acetylglucosamine, NAD^+^, Purine, Uracil, 6-Methyluracil, 5-Methylcytosine, Pseudouridine, 1-Methylguanine
**Carbohydrates**	19	D-Sucrose, Levoglucosan, 1,5-Anhydro-D-glucitol, D-Raffinose, 3-O-β-D-galactosyl-sn-glycerol, Galacturonic acid, L-Threonic acid, 1,3,6-Trigalloyl glucose, Hept-2-ulose, D-Glucono-δ-lactone, 1D-myo-Inositol 1,2-cyclic phosphate, 3,6-Anhydro-β-D-mannopyranose
**Vitamins and cofactors**	8	Dehydroascorbic acid, Pantothenic acid, Pyridoxal, Pyridoxamine, Riboflavin, Thiamine, Nicotinamide, 10-formyldihydrofolic acid
**Terpenes**	5	(+)-Nootkatone, (E,E)-α-Farnesene, (±)-Dihydrojasmonic acid, (−)-trans-Methyl dihydrojasmonate, Tuberonic acid
**Steroids**	1	17,21-Dihydroxypregnenolone
**Flavonoids**	1	Formononetin
**Other (unclassified)**	98	Compounds that could not be confidently assigned to any of the above classes; includes low-abundance or poorly annotated features (full list in Supplementary Table S2)
**Total**	308	

**Table 6:** Number of metabolites detected in ammonium acetate (AA) and sodium nitrate (Nitrate) cultures, and their overlap.

Parameter	Ammonium Acetate (AA)	Sodium Nitrate (Nitrate)
**Total detected metabolites**	250	132
**Common to both sources**	86	86
**Unique to each source**	164	46

## Data Availability

The original data presented in this study are included in the article and its Supplementary Materials. The complete list of identified metabolites, their peak areas, and ^15^N enrichment data are provided in Supplementary Table S1; the lists of unique metabolites for each nitrogen source are provided in Supplementary Table S2. Calibration curves for amino acid quantification and heatmaps of individual amino acid concentrations are provided in Supplementary Figures S1–S19. Further inquiries can be directed to the corresponding author.

## Supplementary Materials

The following supporting information can be downloaded at: https://www.mdpi.com/article/10.3390/ijms27219497/s1.
