# Supplementary material for: Nitrate Versus Ammonium Assimilation in Neochlorella semenenkoi: 15N-Tracking Study of Metabolomic Dynamics
Source: Int J Mol Sci. 2026 Oct 24;27(21):9497. doi: 10.3390/ijms27219497 (PMC13763160; doi:10.3390/ijms27219497)
Supplement: Supplementary file 1 [file ijms-27-09497-s001.zip › Supplementary materials-reversed.pdf]

Supplementary materials for Article

Nitrate versus Ammonium Assimilation in *Neochlorella semenenkoi*: ^{15}N-Tracking Study of Metabolomic Dynamics

Anna Vishnevskaya [1], Maria Sinetova [2], Nikita Malyshev [1], Anastasya Malukova [1], Eugene Nikolaev [3] and Yury Kostyukevich[1]*

1 - Center for Molecular and Cellular Biology, Bolshoy Boulevard 30, bld. 1, Moscow 121205, Russia
2 - K. A. Timiryazev Institute of Plant Physiology, Russian Academy of Sciences, Moscow 121205, Russia
3 - Project Center of Omics Technologies and Advanced Mass Spectrometry, Bolshoy Boulevard 30, bld. 1, Moscow 121205, Russia
* Correspondence: y.kostyukevich@skoltech.ru

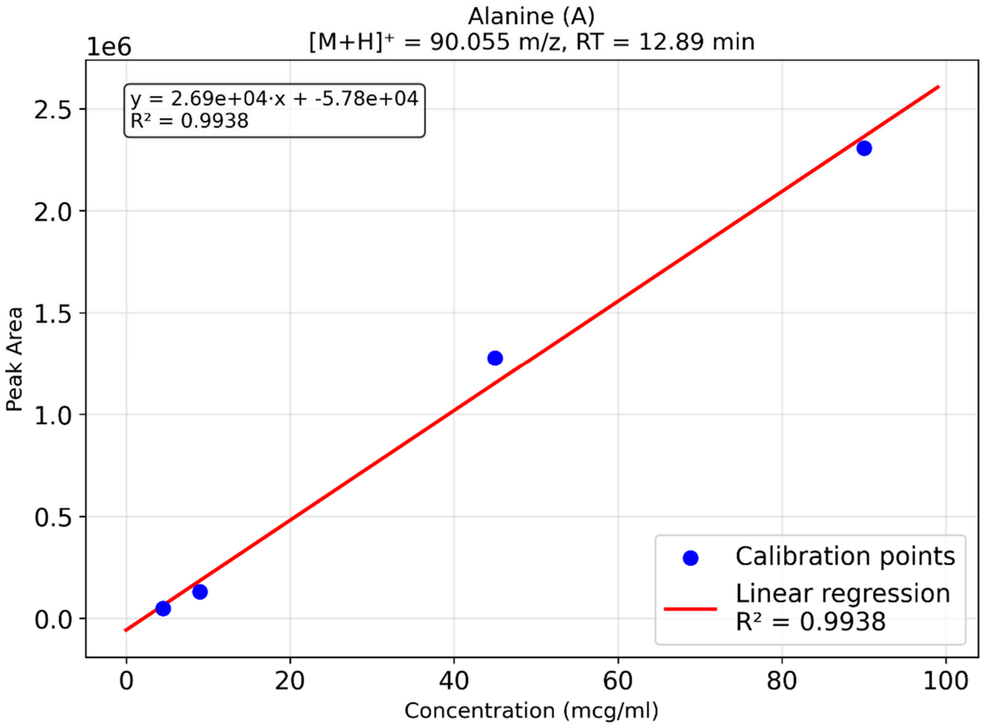

Supplementary Figure S1. Calibration curve for Alanine (A). The curve was generated using external standards in the concentration range 0.009–90 μg/mL. The linear regression equation is $y = 2.69\times10^4 \cdot x - 5.78\times10^4$ with $R^2 = 0.9938$. The curve was used for semi-quantitative determination of alanine in microalgal extracts by HPLC-MS/MS in positive ion mode.

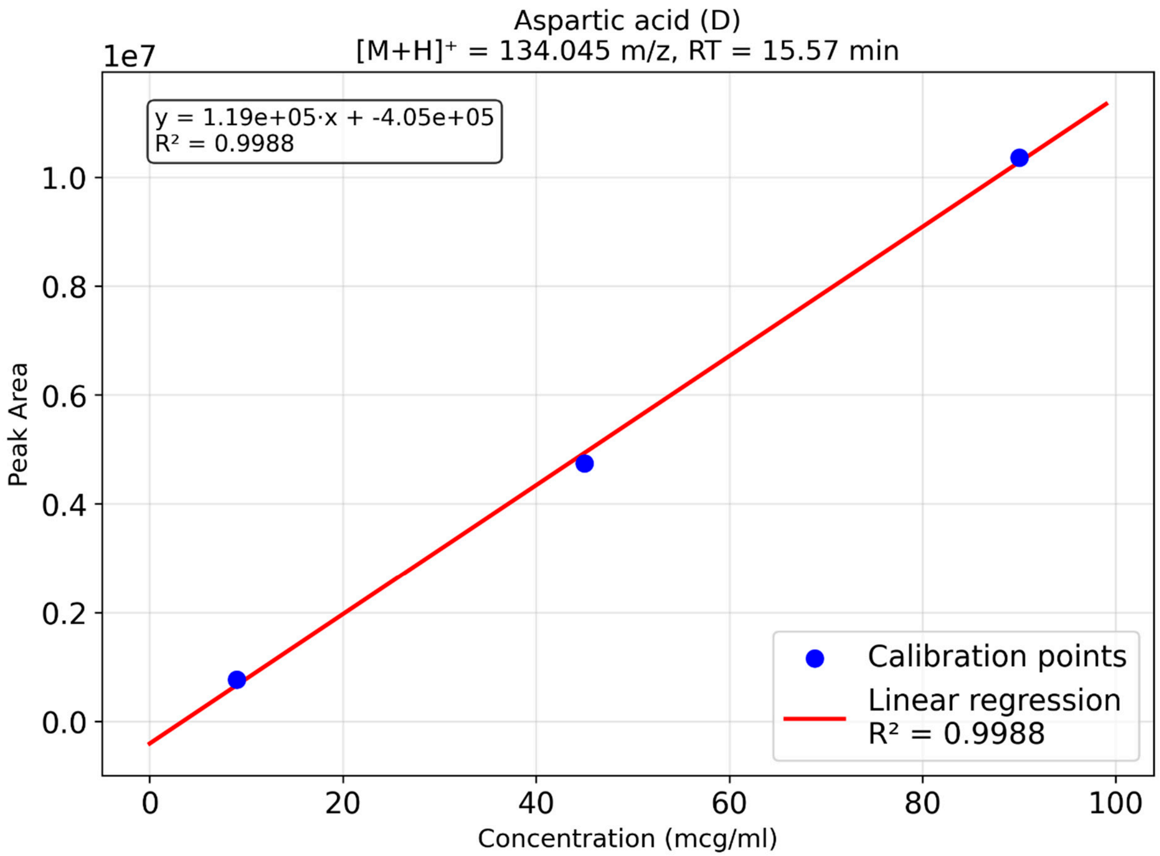

Supplementary Figure S2. Calibration curve for Aspartic acid (D). The curve was generated using external standards in the concentration range 0.009–90 μg/mL. The linear regression equation is $y = 1.19\times10^5 \cdot x - 4.05\times10^5$ with $R^2 = 0.9988$. The curve was used for semi-quantitative determination of aspartic acid in microalgal extracts by HPLC-MS/MS in positive ion mode.

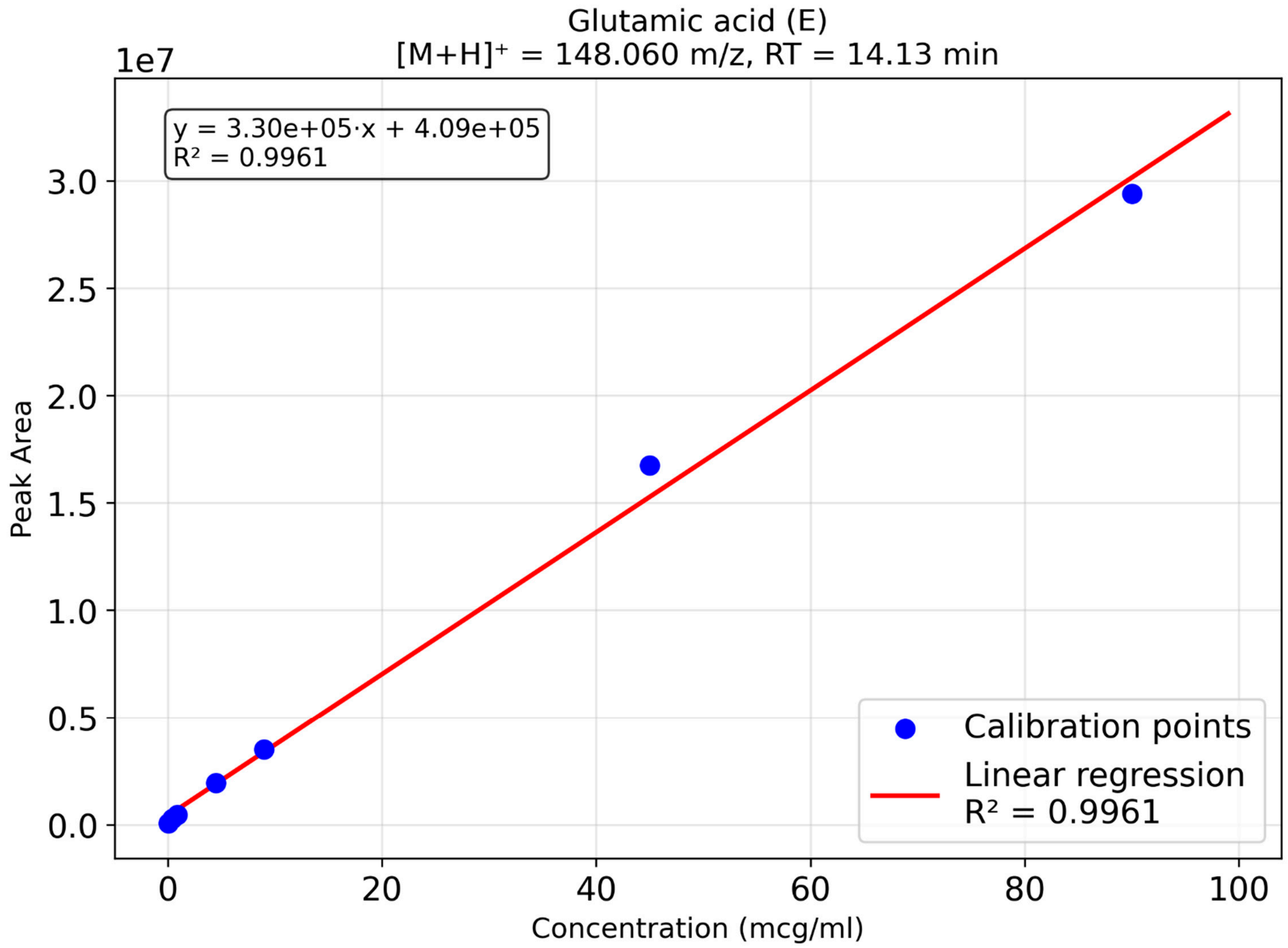

Supplementary Figure S3. Calibration curve for Glutamic acid (E). The curve was generated using external standards in the concentration range 0.009–90 µg/mL. The linear regression equation is $y = 3.30 \times 10^5 \cdot x + 4.09 \times 10^5$ with $R^2 = 0.9961$. The curve was used for semi-quantitative determination of glutamic acid in microalgal extracts by HPLC-MS/MS in positive ion mode.

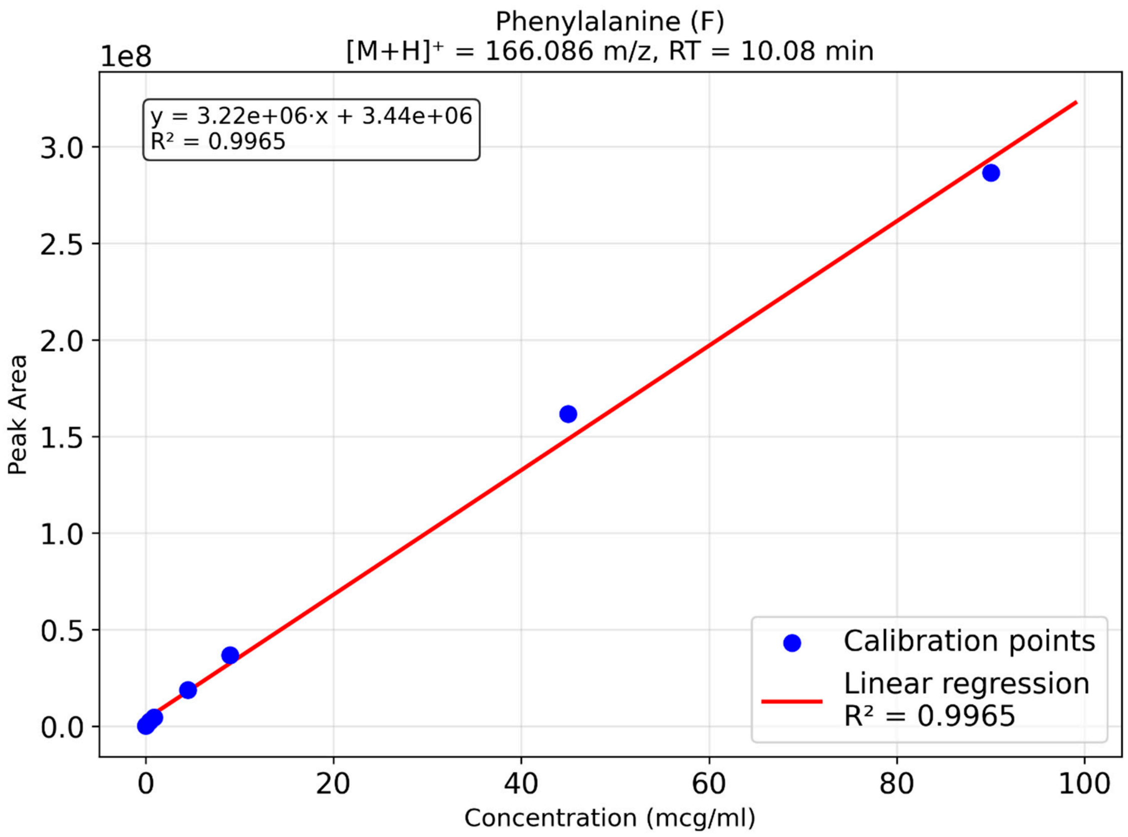

Supplementary Figure S4. Calibration curve for Phenylalanine (F). The curve was generated using external standards in the concentration range 0.009–90 µg/mL. The linear regression equation is $y = 3.22×10^6·x + 3.44×10^6$ with $R^2 = 0.9965$. The curve was used for semi-quantitative determination of phenylalanine in microalgal extracts by HPLC-MS/MS in positive ion mode.

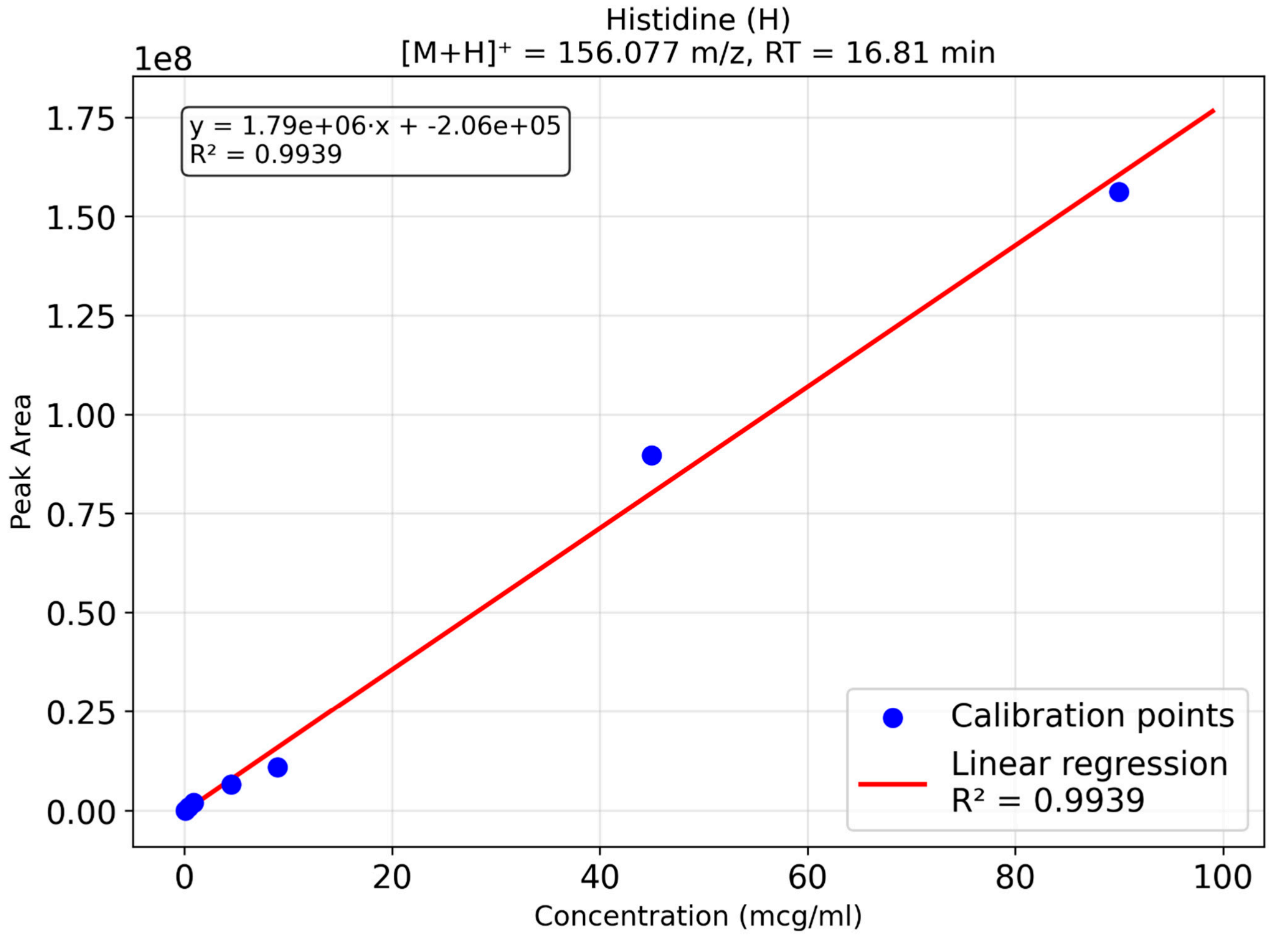

Supplementary Figure S5. Calibration curve for Histidine (H). The curve was generated using external standards in the concentration range 0.009–90 µg/mL. The linear regression equation is $y = 1.79\times10^{6}\cdot x - 2.06\times10^{5}$ with $R^2 = 0.9939$. The curve was used for semi-quantitative determination of histidine in microalgal extracts by HPLC-MS/MS in positive ion mode.

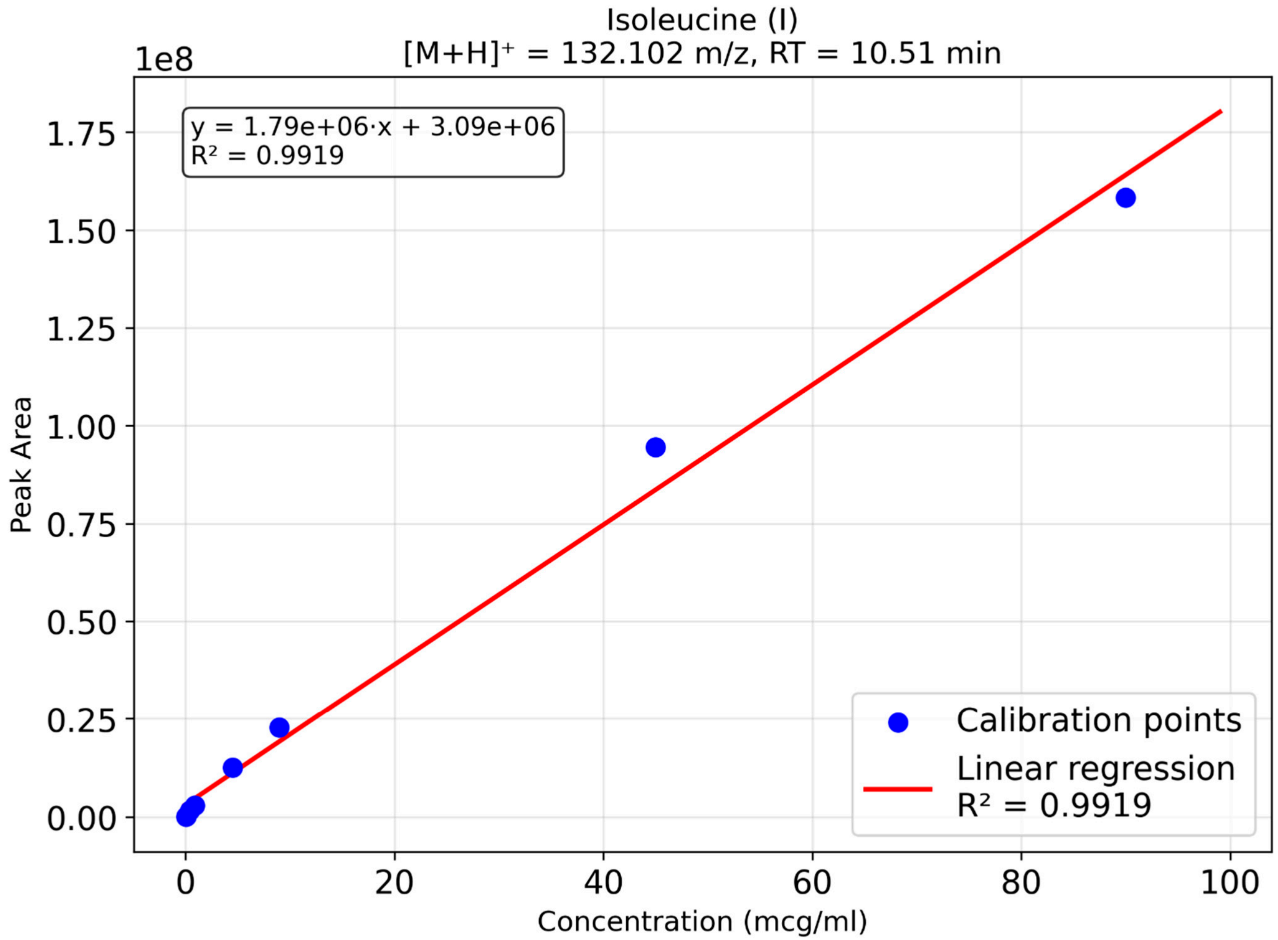

Supplementary Figure S6. Calibration curve for Isoleucine (I). The curve was generated using external standards in the concentration range 0.009–90 μg/mL. The linear regression equation is $y = 1.79{\times}10^6{\cdot}x + 3.09{\times}10^6$ with $R^2 = 0.9916$. The curve was used for semi-quantitative determination of isoleucine in microalgal extracts by HPLC-MS/MS in positive ion mode.

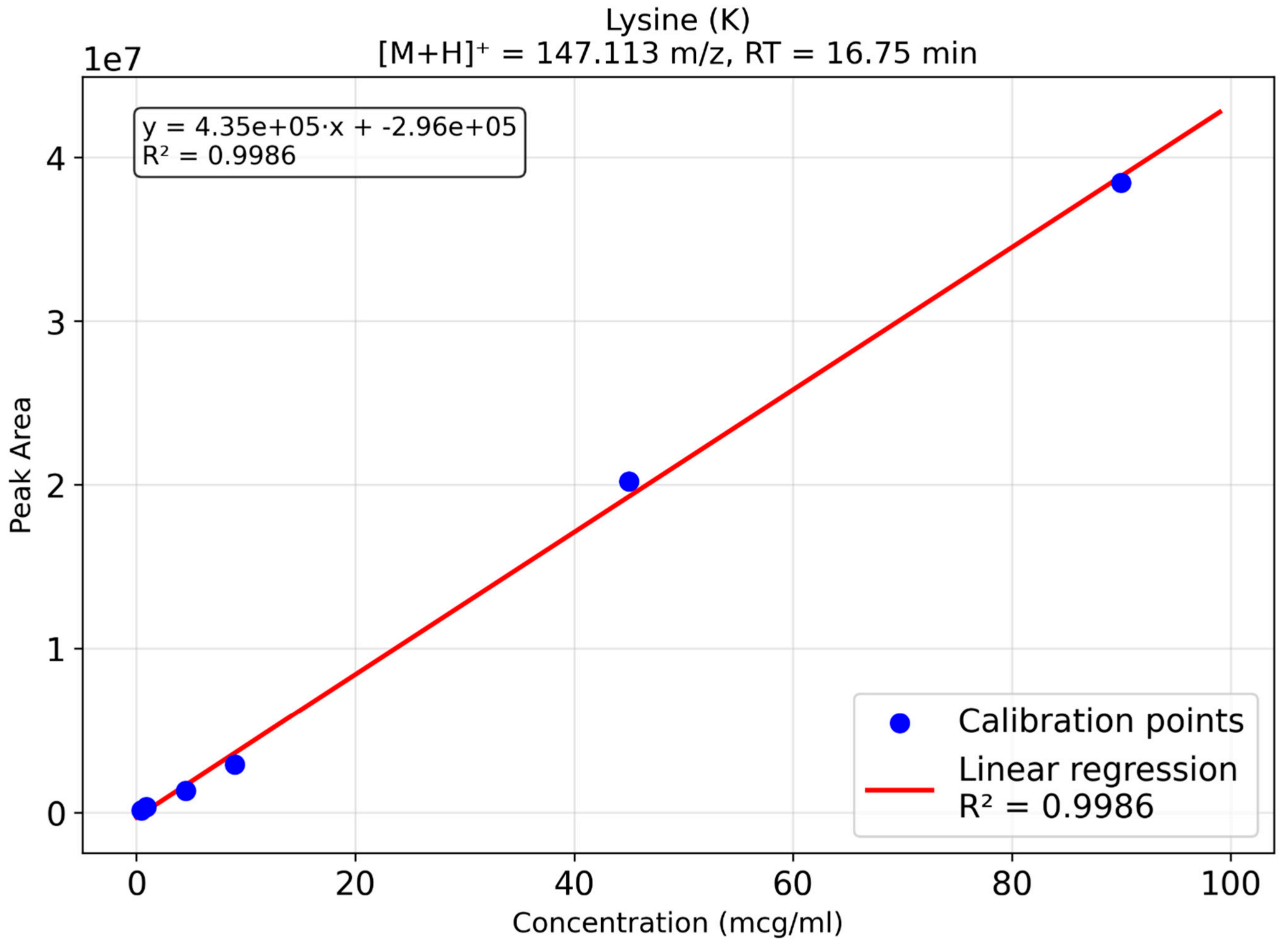

Supplementary Figure S7. Calibration curve for Lysine (K). The curve was generated using external standards in the concentration range 0.009–90 μg/mL. The linear regression equation is $y = 4.35×10^5·x - 2.96×10^5$ with $R^2 = 0.9986$. The curve was used for semi-quantitative determination of lysine in microalgal extracts by HPLC-MS/MS in positive ion mode.

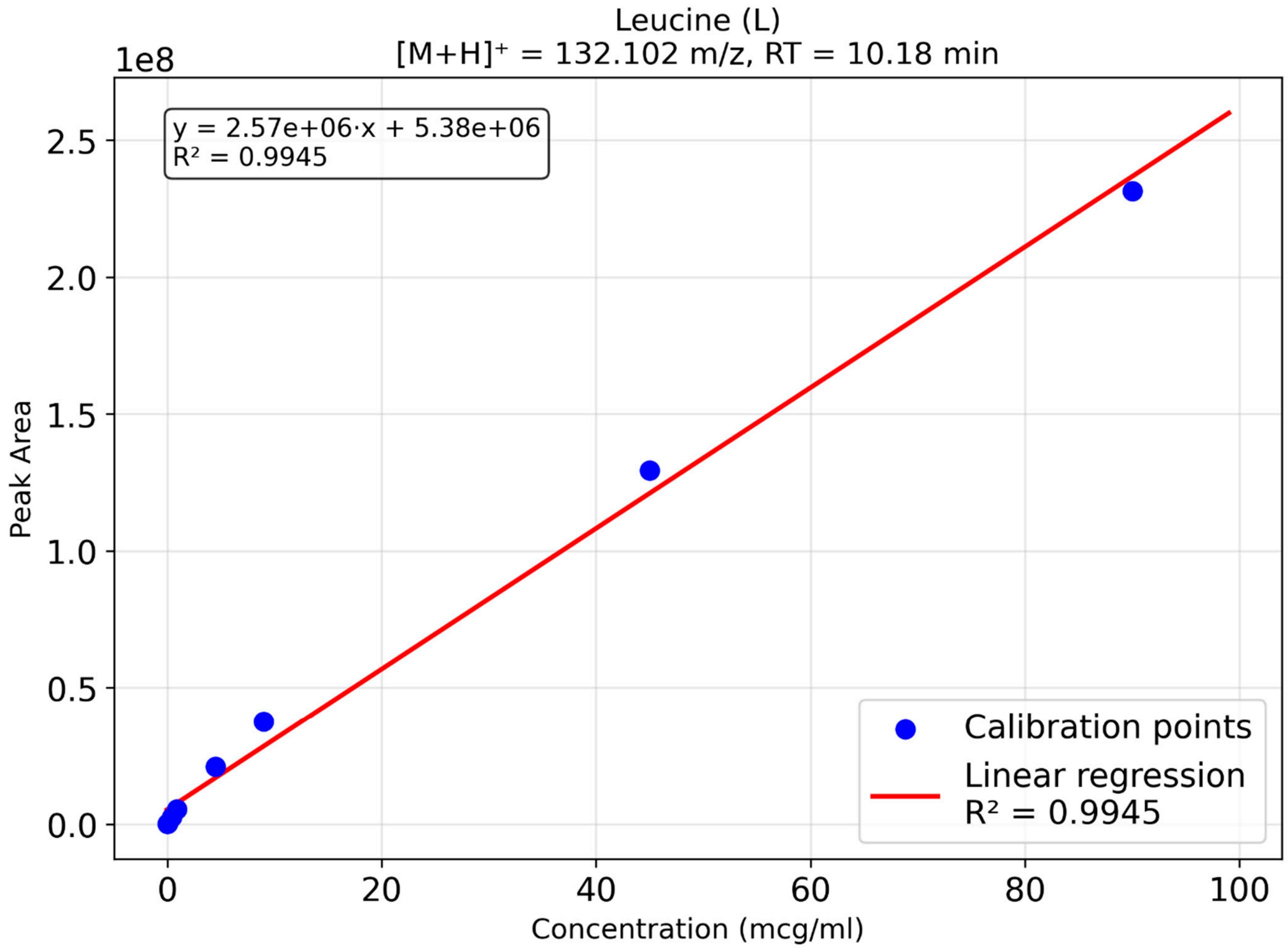

Supplementary Figure S8. Calibration curve for Leucine (L). The curve was generated using the combined peak of leucine and norleucine (internal standard) and corrected accordingly. The concentration range was 0.009–90 μg/mL. The linear regression equation is $y = 2.57 \times 10^6 \cdot x + 5.38 \times 10^6$ with $R^2 = 0.9945$. The curve was used for semi-quantitative determination of leucine in microalgal extracts by HPLC-MS/MS in positive ion mode.

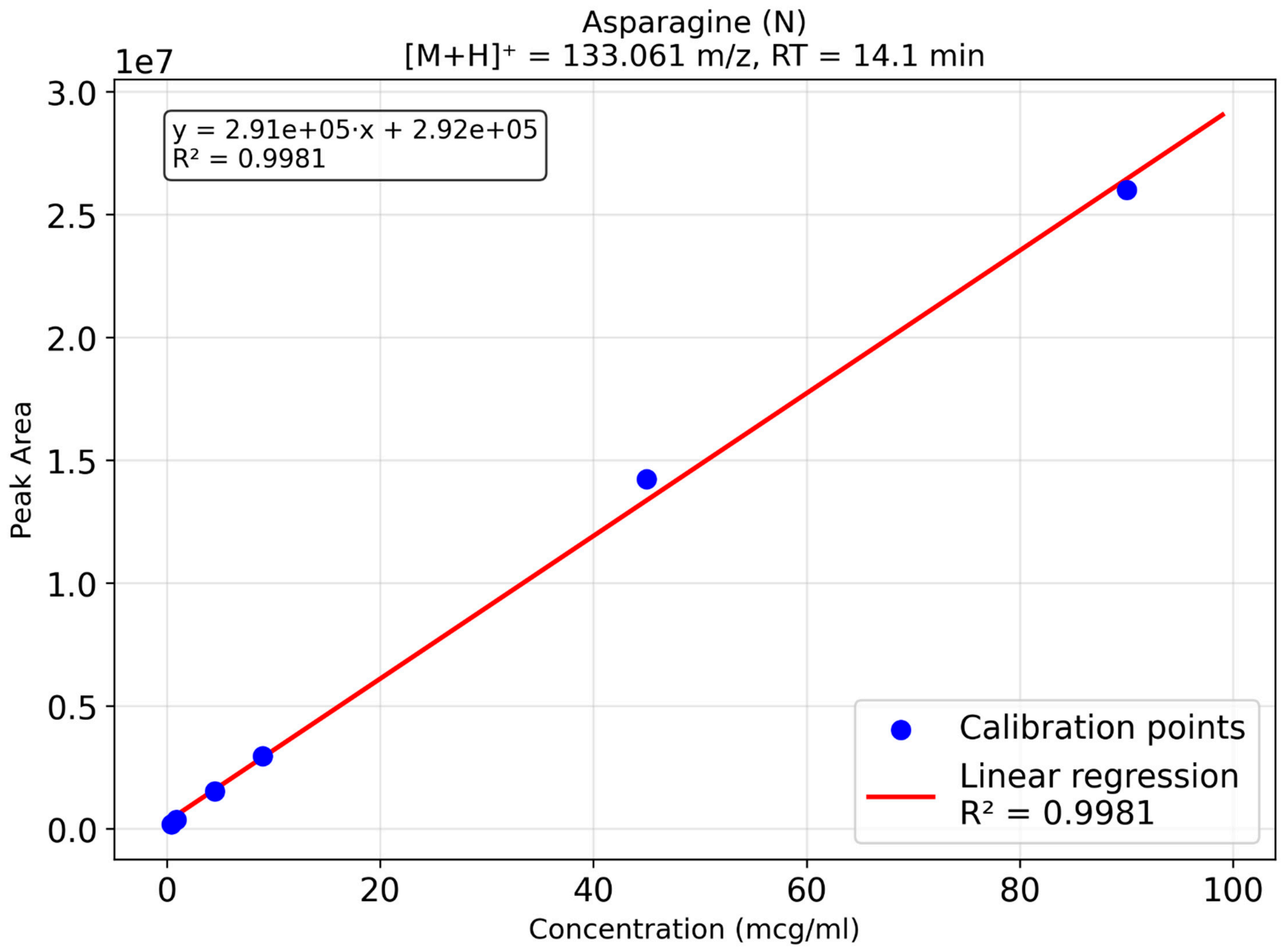

Supplementary Figure S9. Calibration curve for Asparagine (N). The curve was generated using external standards in the concentration range 0.009–90 µg/mL. The linear regression equation is $y = 2.91\times10^5 \cdot x + 2.93\times10^5$ with $R^2 = 0.9981$. The curve was used for semi-quantitative determination of asparagine in microalgal extracts by HPLC-MS/MS in positive ion mode.

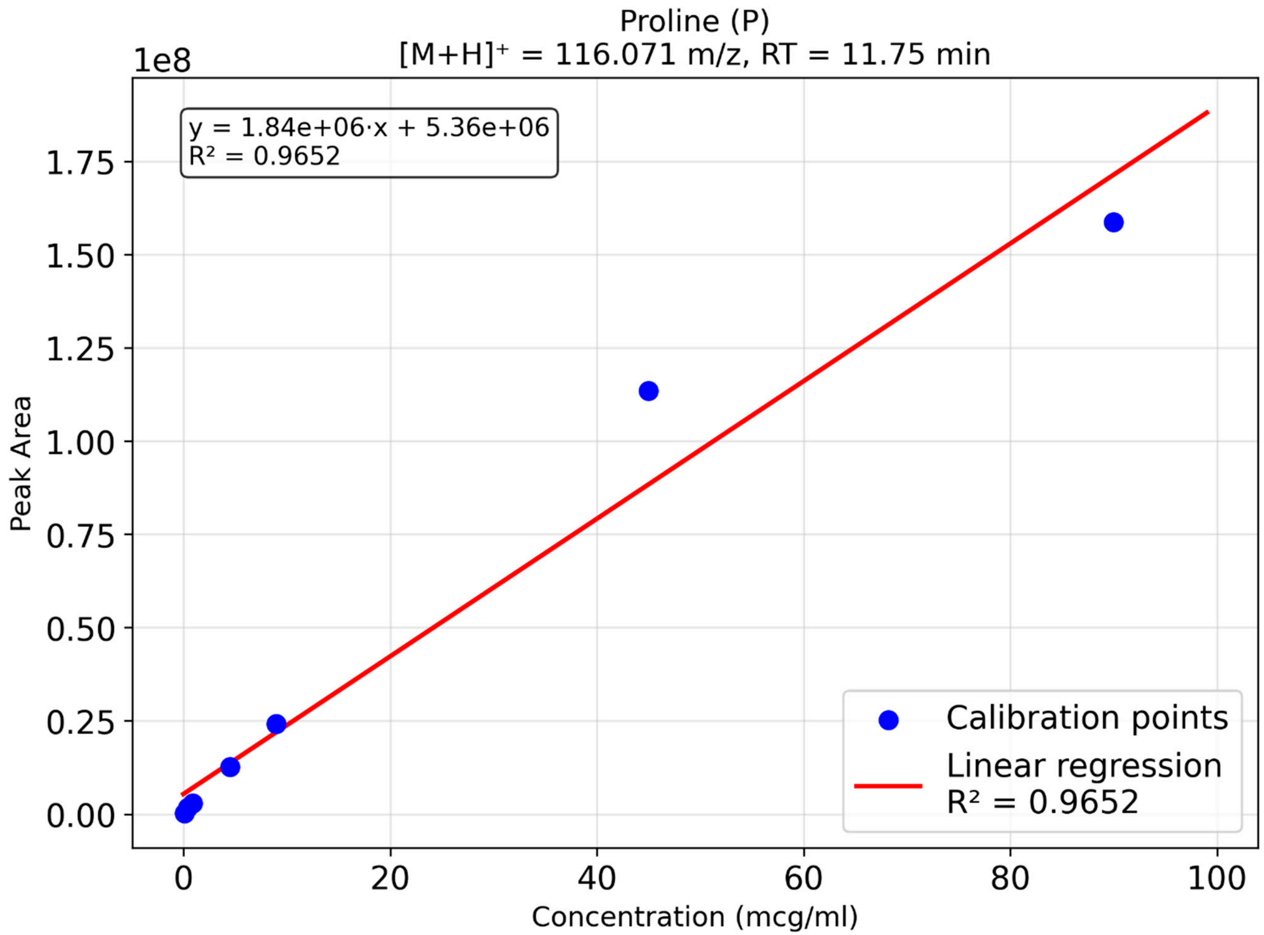

Supplementary Figure S10. Calibration curve for Proline (P). The curve was generated using external standards in the concentration range 0.009–90 µg/mL. The linear regression equation is $y = 1.84×10^6·x + 5.36×10^6$ with $R^2 = 0.9652$. The curve was used for semi-quantitative determination of proline in microalgal extracts by HPLC-MS/MS in positive ion mode.

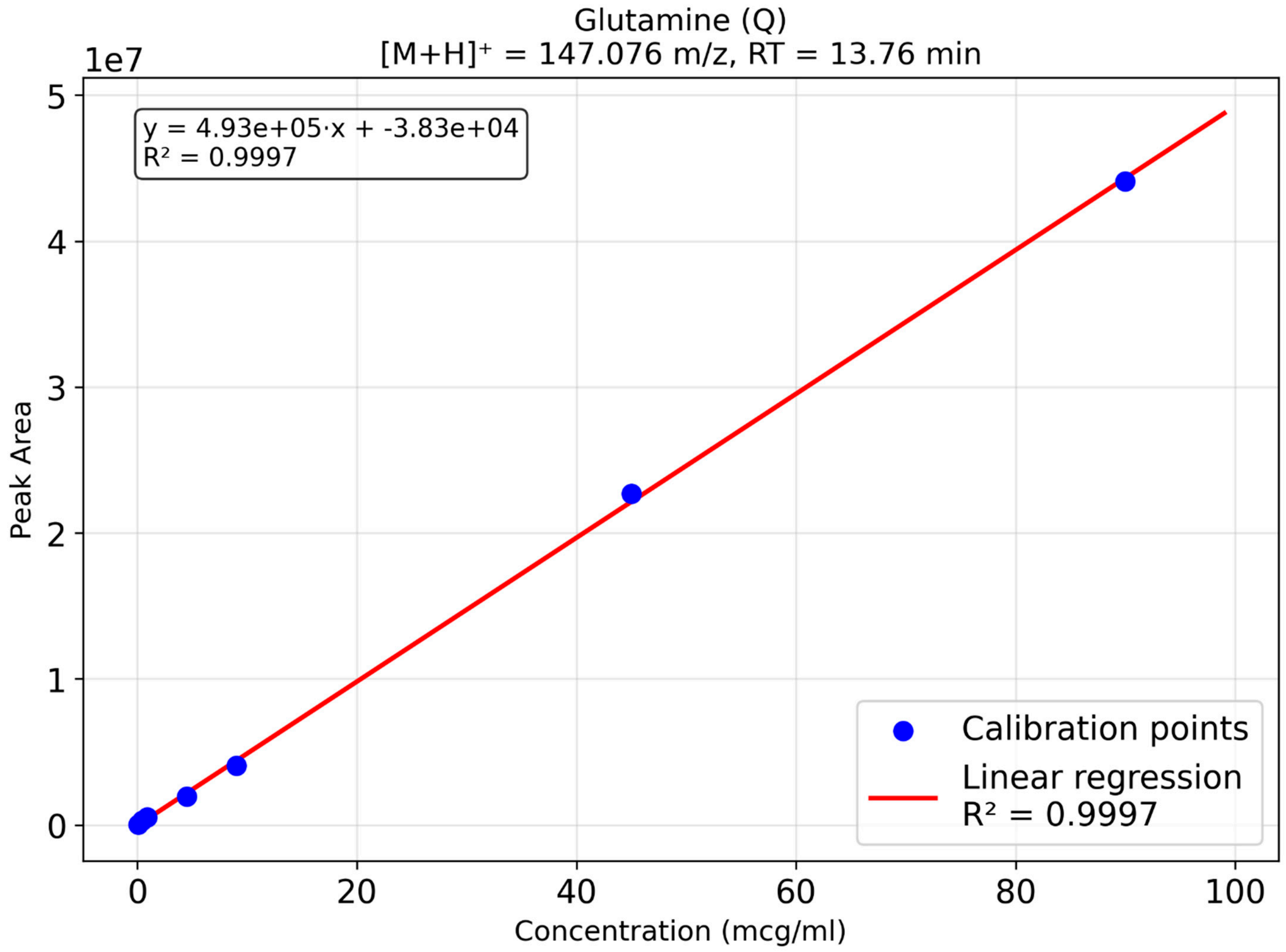

Supplementary Figure S11. Calibration curve for Glutamine (Q). The curve was generated using external standards in the concentration range 0.009–90 µg/mL. The linear regression equation is $y = 4.93\times10^{5}\cdot x + 3.83\times10^{4}$ with $R^2 = 0.9997$. The curve was used for semi-quantitative determination of glutamine in microalgal extracts by HPLC-MS/MS in positive ion mode.

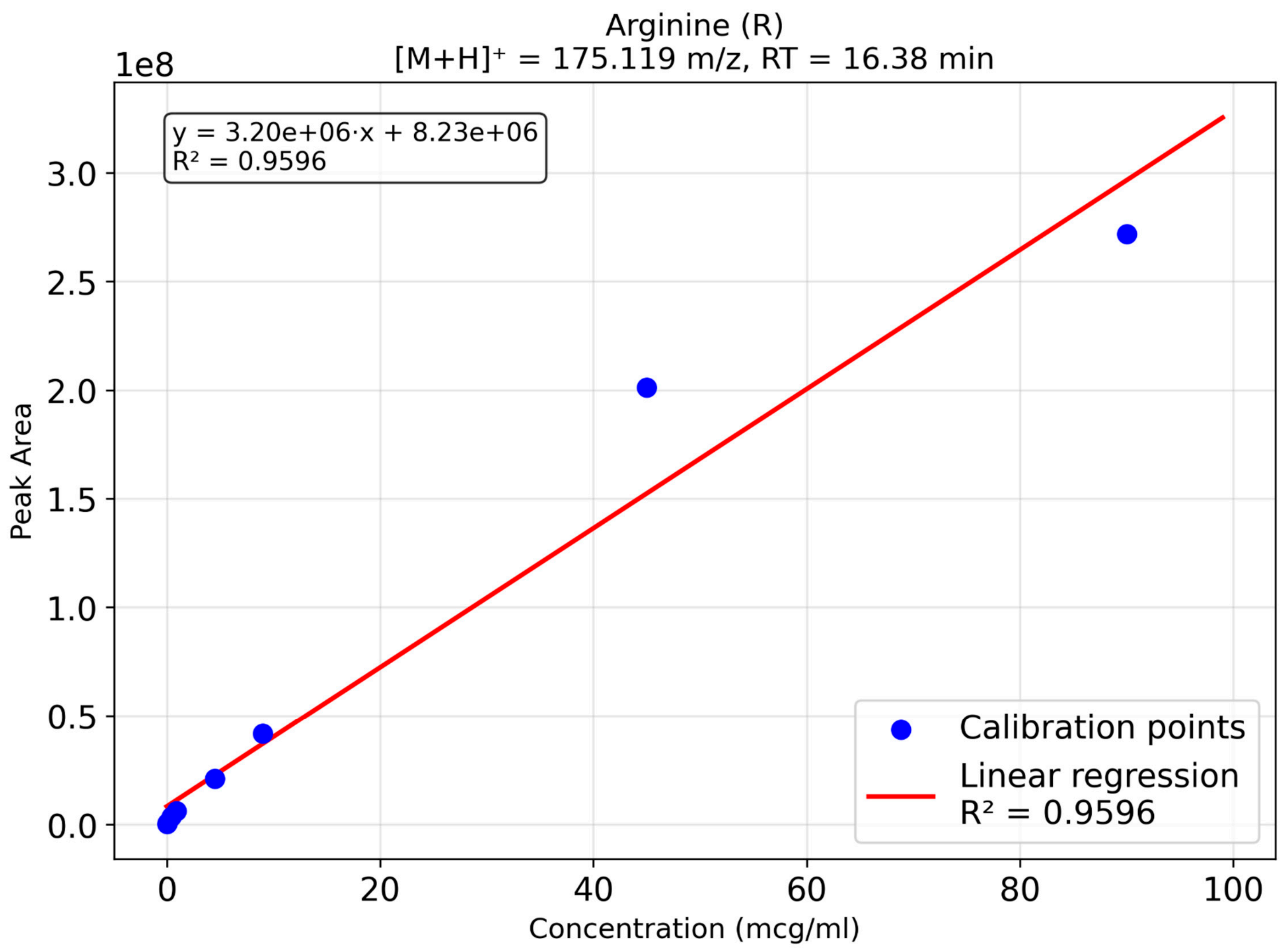

Supplementary Figure S12. Calibration curve for Arginine (R). The curve was generated using external standards in the concentration range 0.009–90 μg/mL. The linear regression equation is $y = 3.20\times10^{6}\cdot x + 8.23\times10^{6}$ with $R^2 = 0.9596$. The curve was used for semi-quantitative determination of arginine in microalgal extracts by HPLC-MS/MS in positive ion mode.

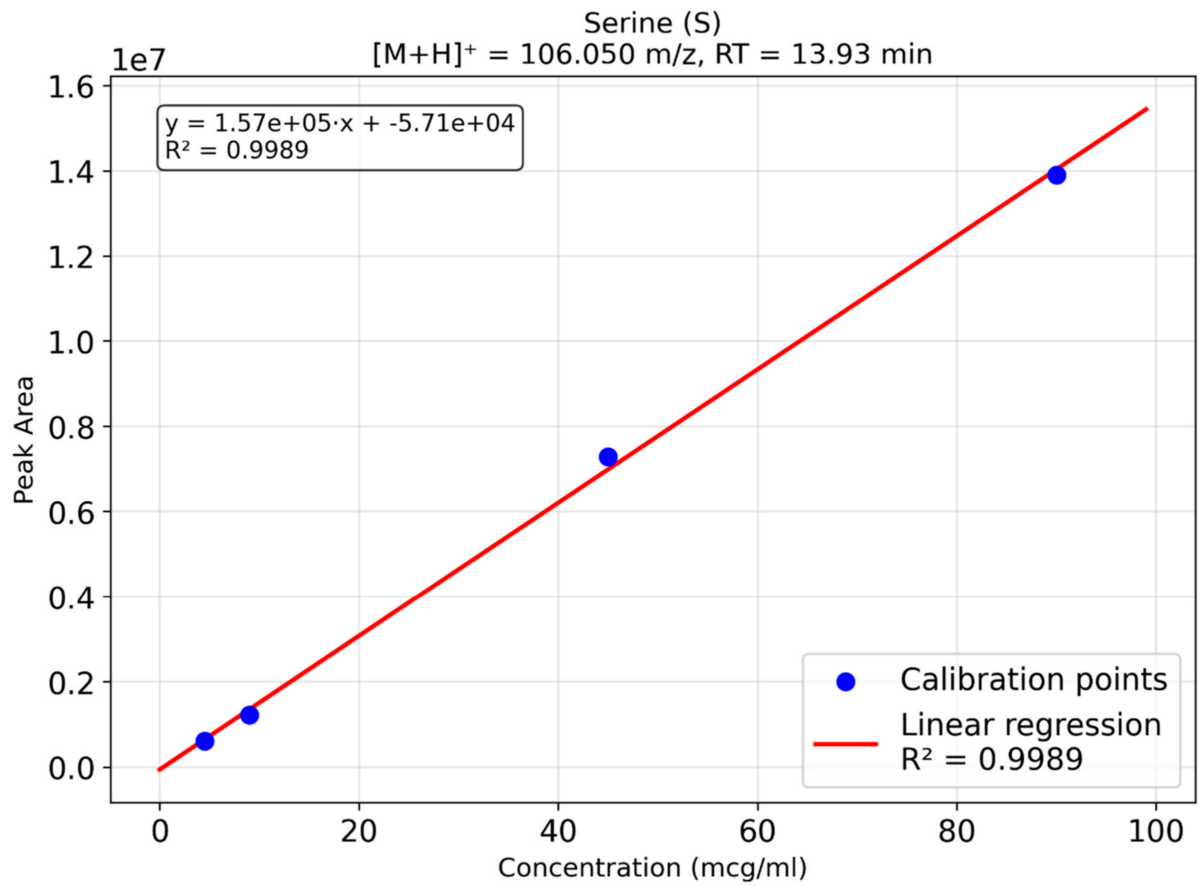

Supplementary Figure S13. Calibration curve for Serine (S). The curve was generated using external standards in the concentration range 0.009–90 μg/mL. The linear regression equation is $y = 1.57{\times}10^5{\cdot}x$ $-5.71{\times}10^4$ with R^2 =0.9989. The curve was used for semi-quantitative determination of serine in microalgal extracts by HPLC-MS/MS in positive ion mode.

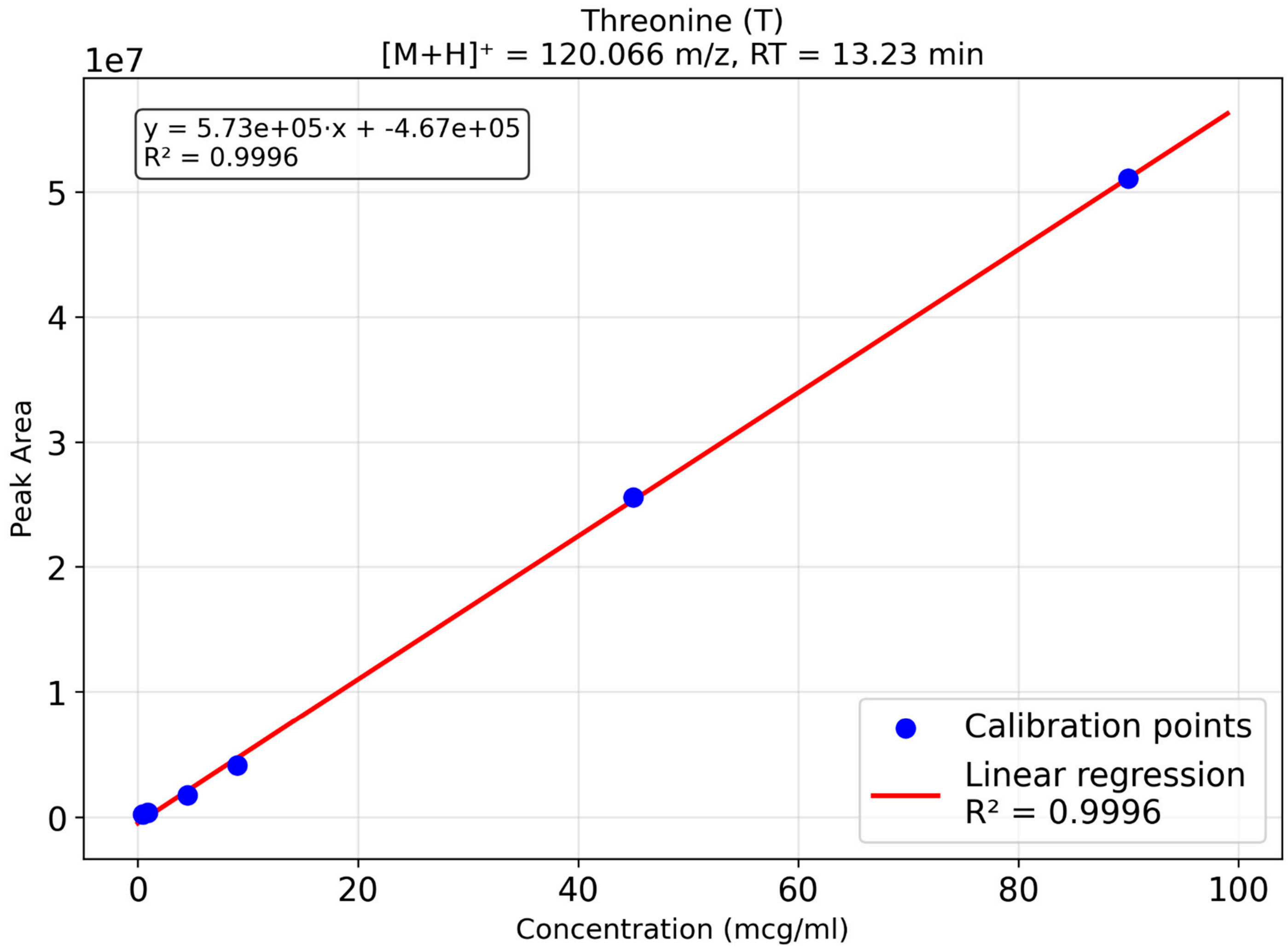

Supplementary Figure S14. Calibration curve for Threonine (T). The curve was generated using external standards in the concentration range 0.009–90 µg/mL. The linear regression equation is $y = 5.73\times10^5\cdot x - 4.67\times10^5$ with $R^2 = 0.9996$. The curve was used for semi-quantitative determination of threonine in microalgal extracts by HPLC-MS/MS in positive ion mode.

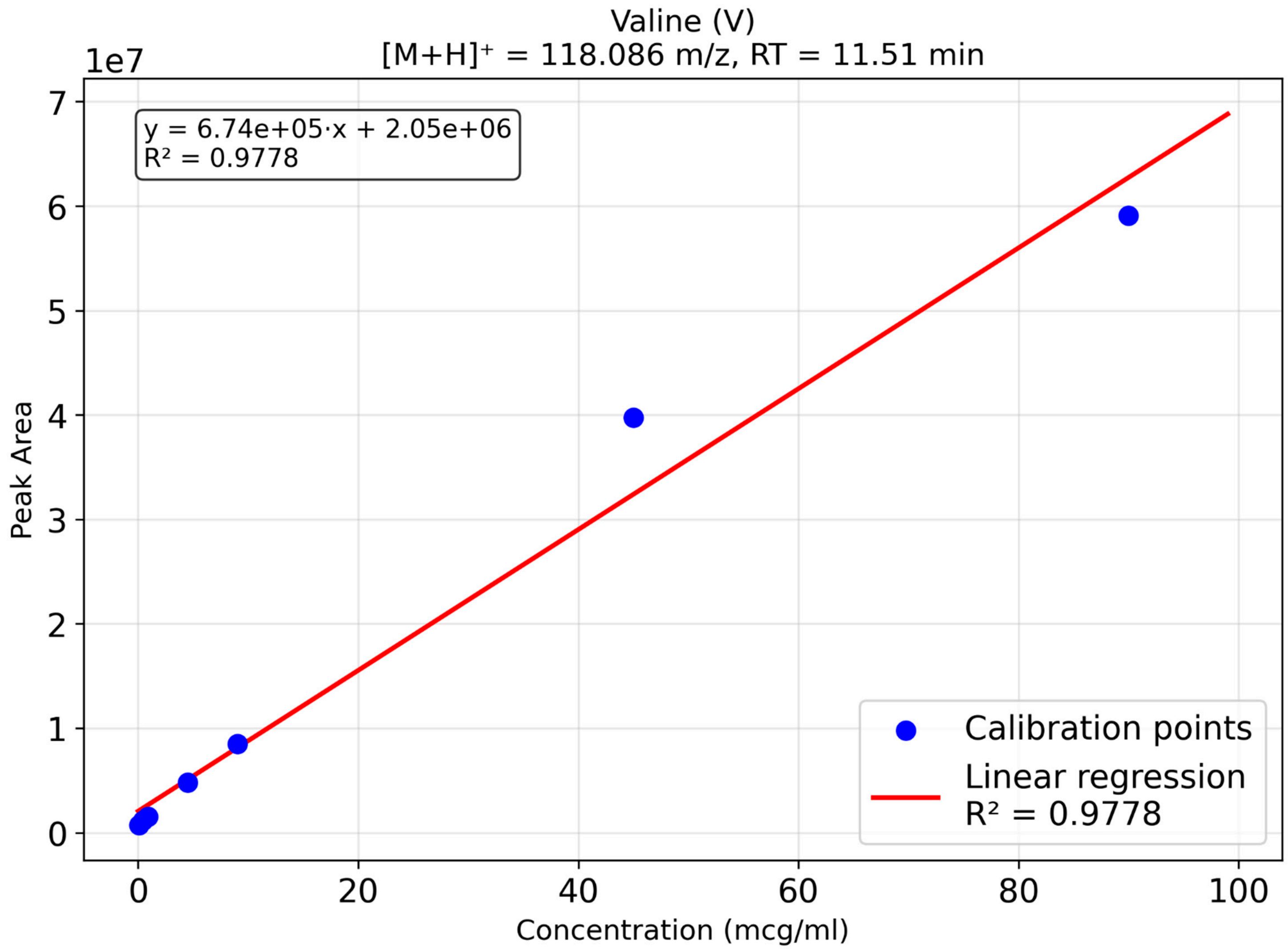

Supplementary Figure S15. Calibration curve for Valine (V). The curve was generated using external standards in the concentration range 0.009–90 μg/mL. The linear regression equation is $y = 6.74\times10^5\cdot x + 2.05\times10^6$ with $R^2 = 0.9778$. The curve was used for semi-quantitative determination of valine in microalgal extracts by HPLC-MS/MS in positive ion mode.

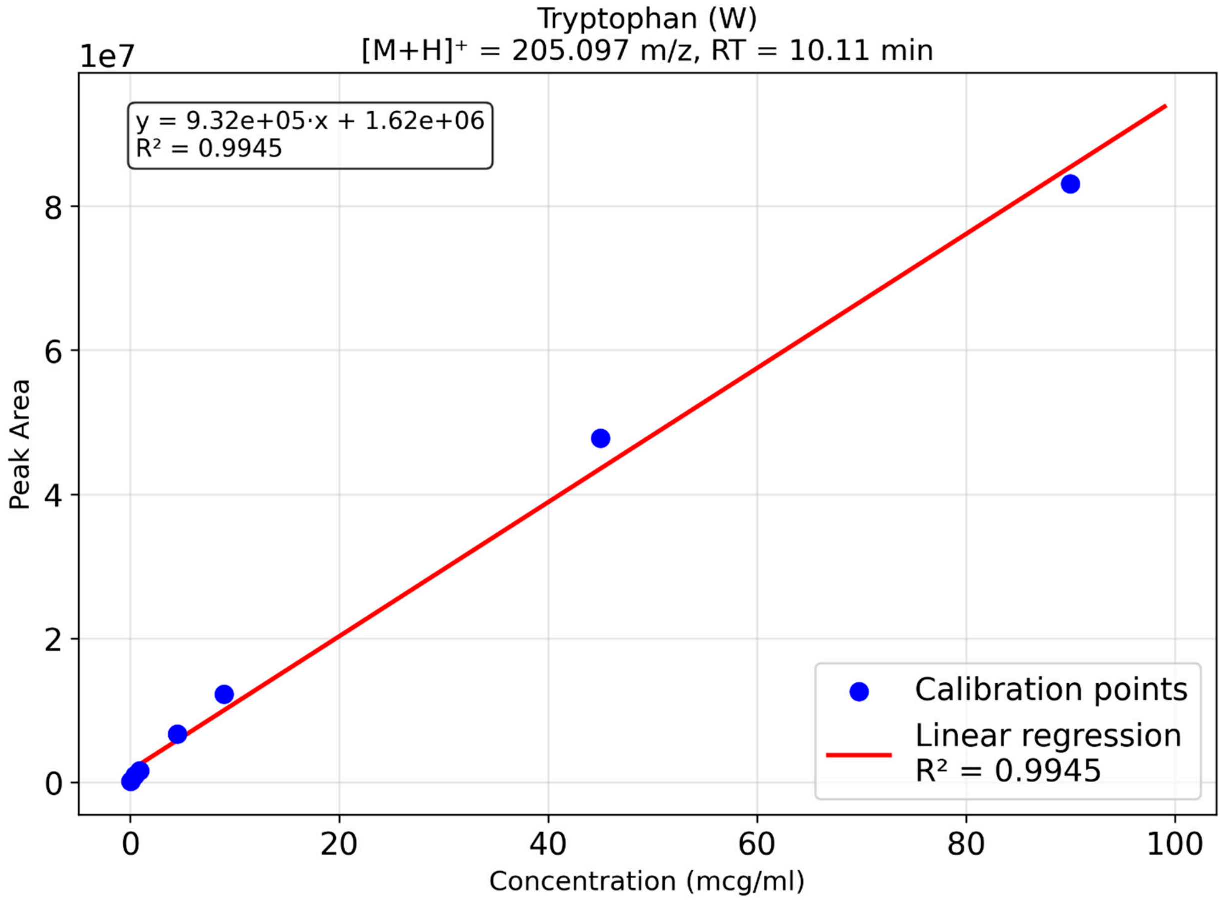

Supplementary Figure S16. Calibration curve for Tryptophan (W). The curve was generated using external standards in the concentration range 0.009–90 μg/mL. The linear regression equation is $y = 9.32 \times 10^5 \cdot x + 1.62 \times 10^6$ with $R^2 = 0.9998$. The curve was used for semi-quantitative determination of tryptophan in microalgal extracts by HPLC-MS/MS in positive ion mode.

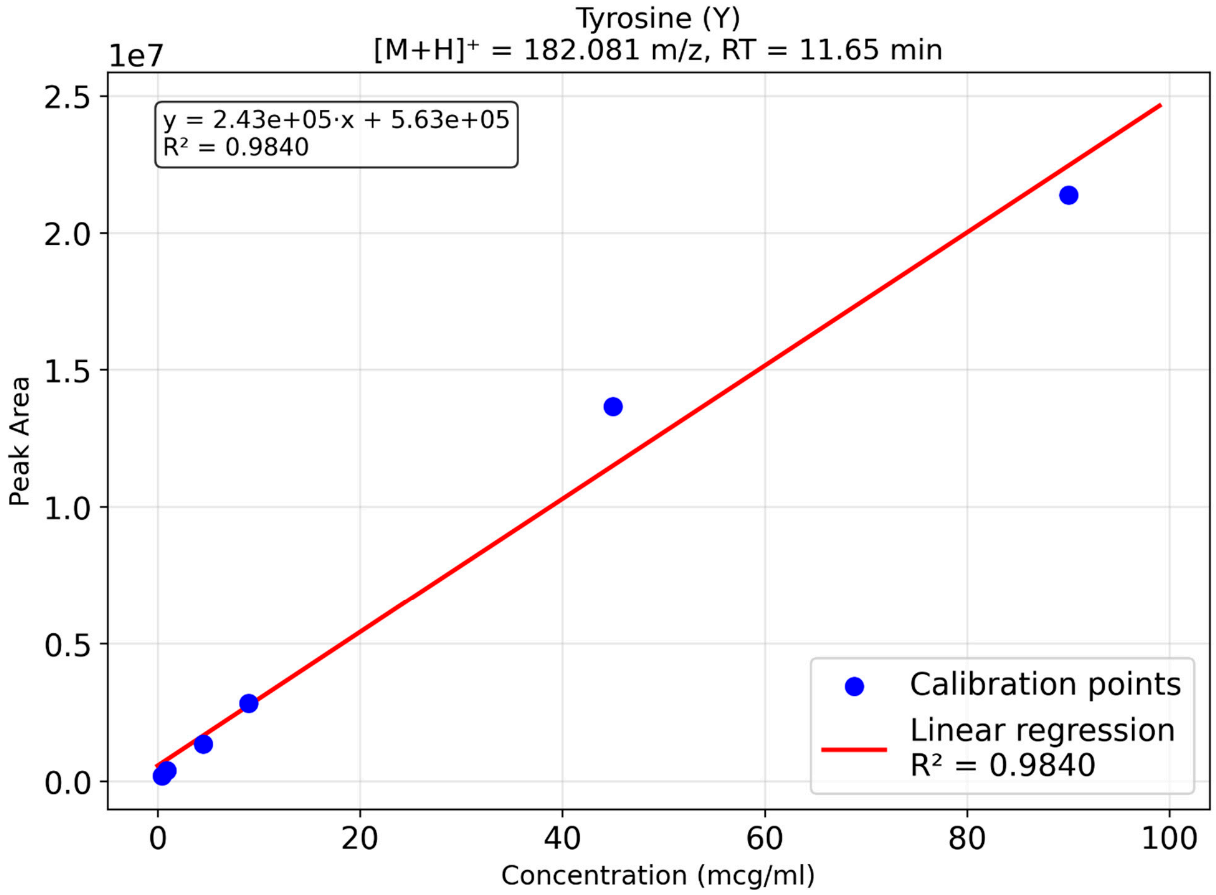

Supplementary Figure S17. Calibration curve for Tyrosine (Y). The curve was generated using external standards in the concentration range 0.009–90 µg/mL. The linear regression equation is $y = 2.43 \times 10^5 \cdot x + 5.63 \times 10^5$ with $R^2 = 0.9840$. The curve was used for semi-quantitative determination of tyrosine in microalgal extracts by HPLC-MS/MS in positive ion mode.

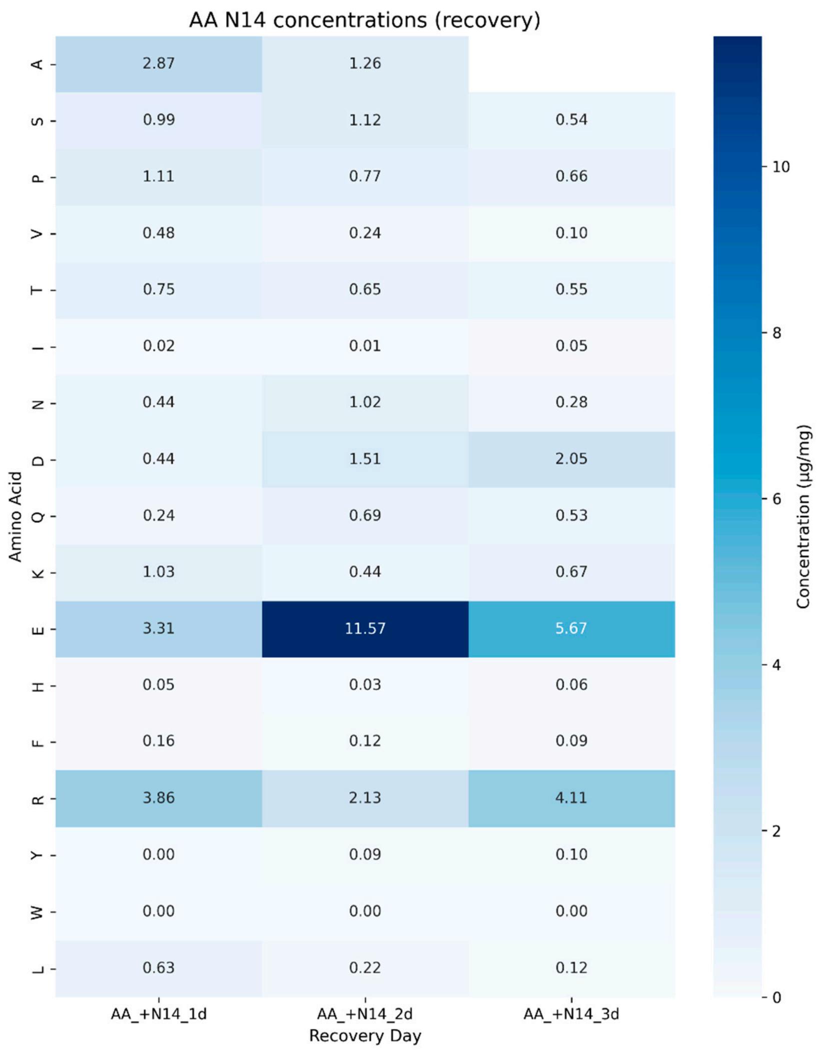

Supplementary Figure S18. Heatmap of amino acid concentrations (µg/mg biomass) in *Neochlorella semenenkoi* IPPAS C-1210 during recovery from nitrogen starvation in ammonium acetate (AA) cultures. Data are shown for the three recovery days (1d, 2d, 3d) after replenishment with unlabeled (^{14}N) ammonium acetate. The color scale ranges from blue (low concentration) to yellow (high concentration). The heatmap provides a visual summary of the absolute concentration profiles for each amino acid, confirming that AA cultures exhibited higher and more uniformly distributed concentrations across amino acids on day 1, with a gradual decline towards day 3. Glutamate, glutamine, arginine, and threonine showed consistently high levels throughout the recovery period.

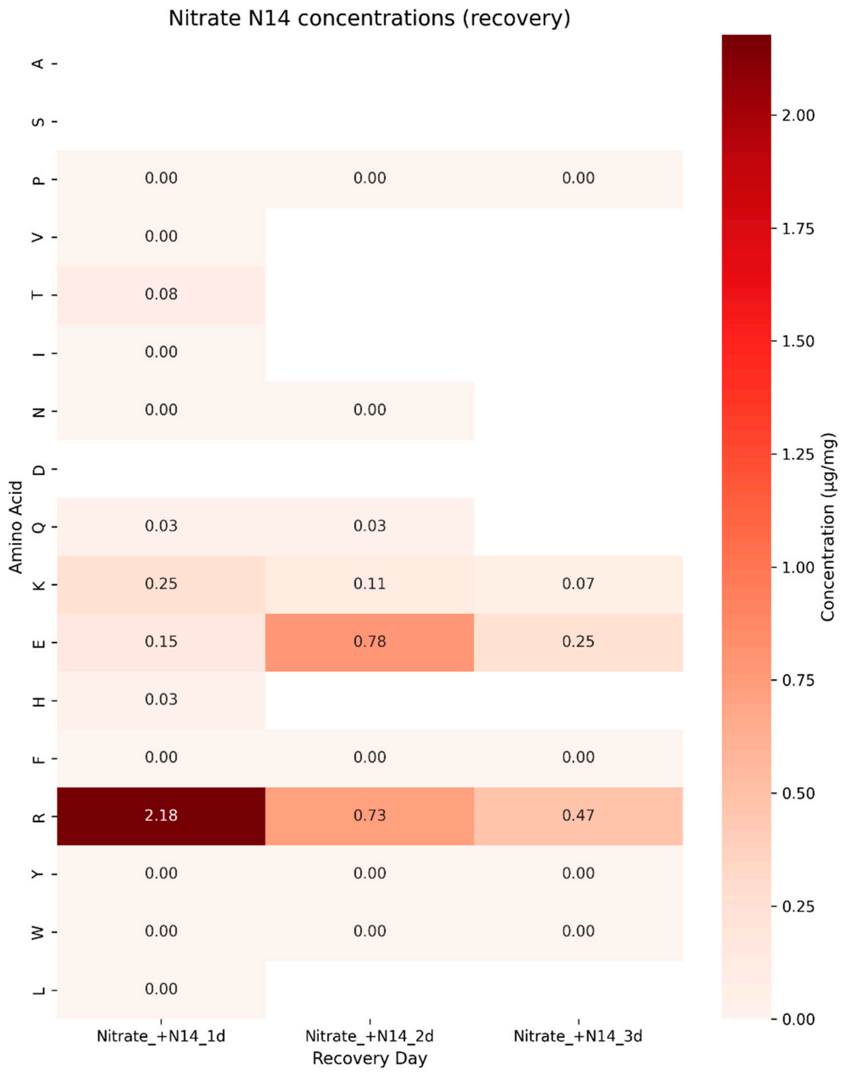

Supplementary Figure S19. Heatmap of amino acid concentrations (µg/mg biomass) in *Neochlorella semenenkoi* IPPAS C-1210 during recovery from nitrogen starvation in sodium nitrate (Nitrate) cultures. Data are shown for the three recovery days (1d, 2d, 3d) after replenishment with unlabeled (^{14}N) sodium nitrate. The color scale ranges from blue (low concentration) to yellow (high concentration). The heatmap illustrates that nitrate cultures exhibited very low or undetectable levels for many amino acids on day 1, with gradual recovery by day 3, consistent with the slower assimilation kinetics of nitrate. By day 3, the concentration patterns became similar to those observed in AA cultures.
